# Recent Progress in the Sustainable Synthesis of Imidazole Derivatives: Advances in Multicomponent Reactions, Catalysis, and Green Chemistry

**DOI:** 10.3390/molecules31142439

**Published:** 2026-07-11

**Authors:** Altynay B. Kaldybayeva, Timur T. Shapinov, Feyyaz Durap, Valentina K. Yu

**Affiliations:** 1 Department of Chemistry and Technology of Organic Substances, Natural Compounds and Polymers, Al-Farabi Kazakh National University, Almaty 050040, Kazakhstan; 2 Laboratory of Chemistry of Synthetic and Natural Medicinal Compounds, A.B. Bekturov Institute of Chemical Sciences, Almaty 050010, Kazakhstan; 3 School of Chemical Engineering, Kazakh-British Technical University, Almaty 050000, Kazakhstan; ttshapka@gmail.com; 4 Department of Chemistry, Dicle University, Diyarbakir 21280, Turkey; feyyazdurap@gmail.com

**Keywords:** imidazole derivatives, multicomponent reactions, Debus–Radziszewski reaction, green synthesis, catalysis, heterocycles

## Abstract

Imidazole derivatives represent a privileged class of nitrogen-containing heterocycles that continue to attract considerable attention owing to their structural versatility, synthetic accessibility, and wide range of applications in medicinal chemistry, materials science, catalysxis, and corrosion inhibition. In recent years, significant efforts have been directed towards the development of efficient, sustainable, and experimentally simple synthetic methodologies for substituted imidazoles. This review summarizes and critically evaluates recent advances (mainly from 2021 onward) in the synthesis of imidazole derivatives, with particular emphasis on multicomponent reactions, especially the Debus–Radziszewski condensation. Catalyst-free protocols, organocatalysts, metal-based catalysts, nanocatalysts, and ionic liquids are systematically discussed and compared. In addition, alternative two- and three-component strategies that enable access to nonclassical substitution patterns are reviewed. The advantages and limitations of each approach are highlighted with respect to reaction efficiency, substrate scope, sustainability, and practical applicability. This review aims to serve as a comprehensive reference for synthetic chemists seeking rational strategies for the preparation of structurally diverse imidazole derivatives.

## 1. Introduction

Imidazole derivatives constitute a privileged class of nitrogen-containing heterocycles and remain among the most intensively investigated scaffolds in contemporary organic chemistry. The imidazole ring, featuring two nonequivalent nitrogen atoms, combines aromatic stability with pronounced electronic and hydrogen-bonding flexibility, which accounts for its broad applicability across multiple scientific domains.

According to Scopus, more than 3000 publications on imidazole synthesis have appeared since 2020. Numerous imidazole-based compounds exhibit antiparasitic [1,2], antimicrobial [3,4,5,6,7,8,9,10,11,12,13,14,15,16,17,18,19], antifungal, anticancer [20,21,22,23,24,25,26,27,28,29,30,31,32,33,34,35,36,37], antiviral, and anti-inflammatory activities [38,39,40]. In addition to medicinal chemistry, imidazole derivatives have found applications as corrosion inhibitors [41,42,43,44], functional organic materials, ionic liquids, and ligands for coordination and catalytic systems [45]. The coexistence of biological relevance and physicochemical versatility highlights imidazole as a multifunctional molecular platform [46,47]. The rapid expansion of synthetic methodologies together with the growing importance of imidazole derivatives in medicinal and materials chemistry has stimulated significant research activity in this field.

The continuous demand for structurally diverse imidazole derivatives has stimulated the development of efficient and sustainable synthetic methodologies. In this context, multicomponent reactions (MCRs) have emerged as particularly powerful tools, enabling the rapid assembly of complex imidazole frameworks from simple starting materials. Among these approaches, the Debus–Radziszewski reaction remains the most widely employed and versatile strategy [48].

**Figure 1:**
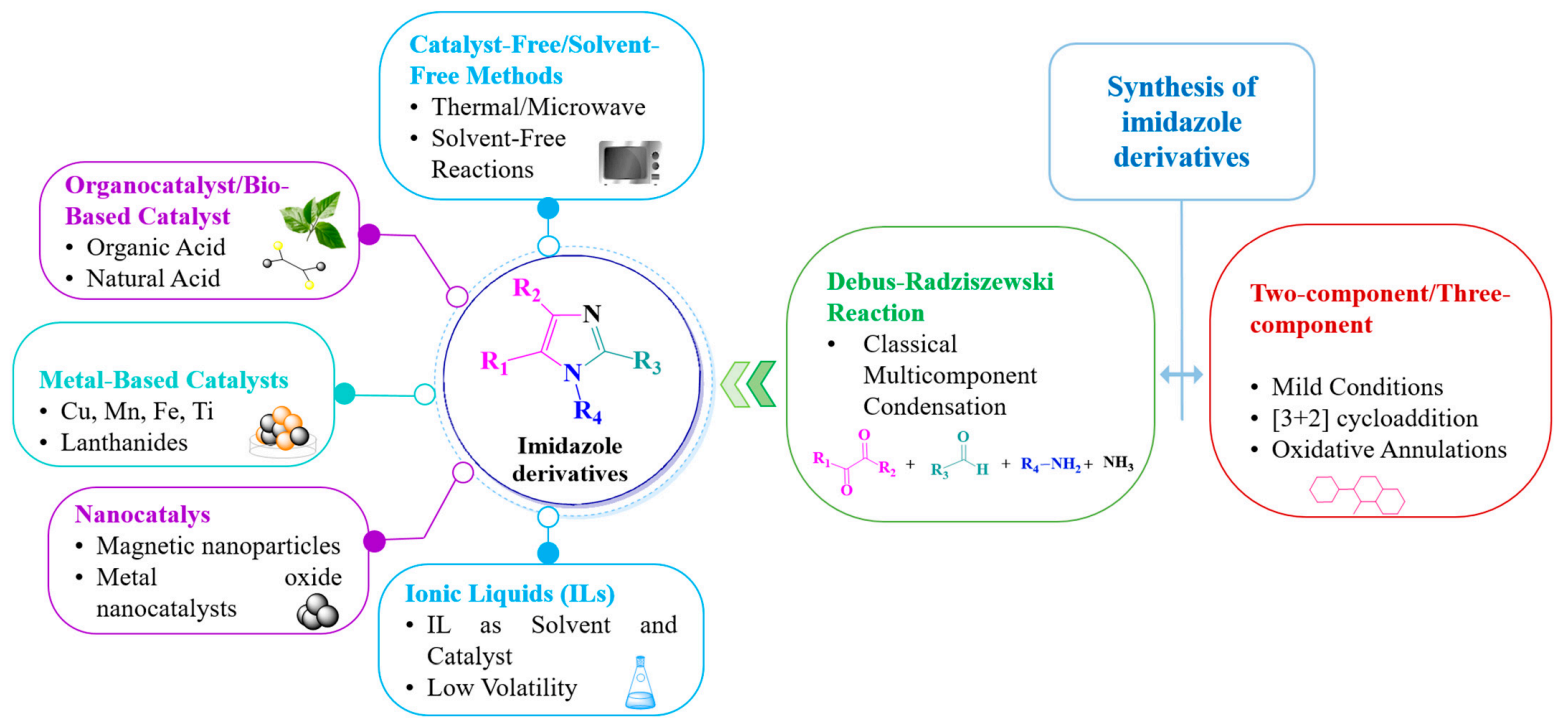
Synthetic approaches to imidazole synthesis.

A graphical representation of the main synthetic strategies discussed in this review is provided in Figure 1. These methods collectively demonstrate the evolution of imidazole synthesis, from classical condensation reactions to more efficient, environmentally friendly, and versatile catalytic systems.

Overview of the major synthetic strategies employed for the preparation of imida-zole derivatives, encompassing the classical Debus–Radziszewski multicomponent condensation, catalyst-free and solvent-free methodologies, transition metal catalysis, nanocatalytic systems, organocatalysis, bio-based catalysts, ionic liquid media, and alternative two- and three-component approaches. The figure highlights the diversification of catalytic platforms and the growing emphasis on sustainable, recyclable, and environmentally benign methodologies in modern imidazole synthesis.

## 2. Multicomponent Synthesis via the Debus–Radziszewski Reaction

### 2.1. General Aspects

**Scheme 1:**
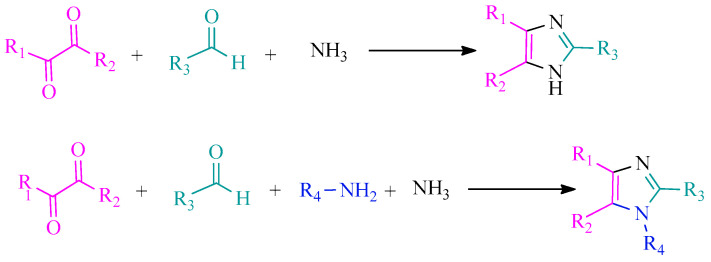
General scheme of the Debus–Radziszewski multicomponent synthesis of substituted imidazoles.

The Debus–Radziszewski reaction is a classical multicomponent condensation involving a 1,2-dicarbonyl compound, an aldehyde, and a nitrogen source such as ammonia, ammonium salts, or primary amines, leading to tri- or tetrasubstituted imidazoles (Scheme 1) [49].

Graphical overview of the major synthetic strategies for the preparation of imidazole derivatives, including classical Debus–Radziszewski condensation, catalytic methodologies, nanocatalysis, ionic liquids, and alternative annulation reactions.

### 2.2. Catalyst-Free and Solvent-Free Approaches

Recent studies have demonstrated that the Debus–Radziszewski reaction can proceed efficiently under catalyst-free conditions using thermal, microwave (M.W.), or ultrasonic (US) activation. Such approaches reduce waste generation and simplify purification procedures, which is consistent with the principles of green chemistry. In addition, the absence of metal-based or organocatalysts decreases the environmental impact and eliminates challenges related to catalyst recovery and deactivation. Non-conventional activation methods can also enhance reaction efficiency by accelerating reaction rates and improving reproducibility. As a result, these strategies provide practical and scalable alternatives for the synthesis of imidazole derivatives and highlight the increasing role of sustainable methodologies in modern heterocyclic synthesis [50,51].

**Scheme 2:**
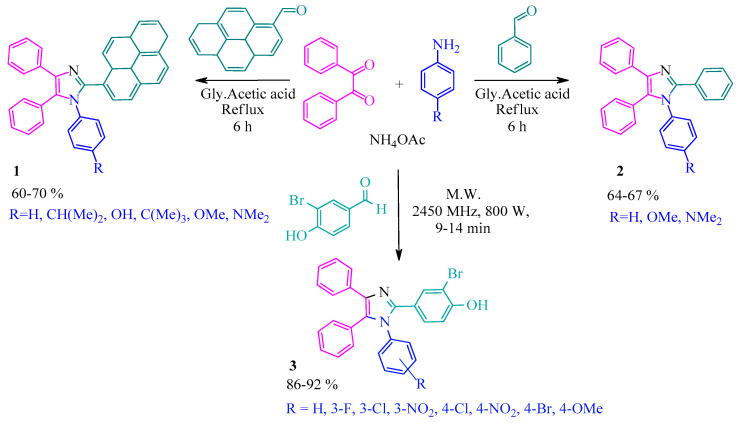
Catalyst-free multicomponent synthesis of 1,2,4,5–substituted imidazole derivatives.

The synthesis of nine 1,2,4,5-tetrasubstituted imidazole derivatives **1**, **2** via a catalyst-free four-component reaction demonstrates a synthetically straightforward approach (Scheme 2); however, the reliance on glacial acetic acid as both solvent and reaction medium may limit broader applicability due to its harsh and non-green nature. While the use of diverse aniline derivatives and two distinct aldehydes (benzaldehyde and pyrene aldehyde) highlights structural versatility, the average isolated yield of 67% suggests only acceptable efficiency compared to more optimized catalytic systems reported in the literature. Furthermore, the absence of a catalyst, although advantageous from a simplicity standpoint, raises questions regarding reaction selectivity and scalability, particularly for electron-rich or sterically hindered substrates. Overall, while the methodology provides access to functionalized imidazoles relevant for OLED applications, further optimization would be necessary to improve sustainability and synthetic performance [52].

**Scheme 3:**
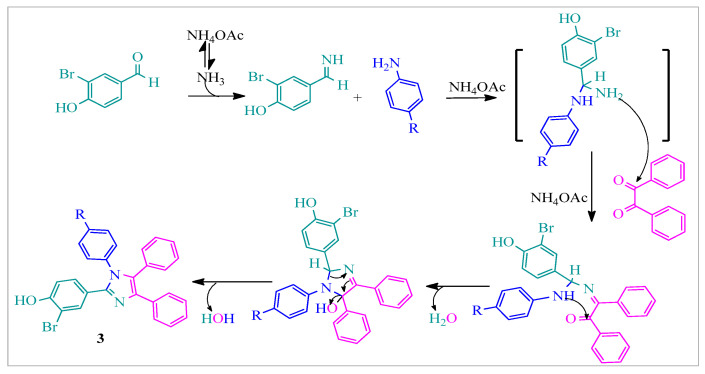
Proposed reaction mechanism for the formation of 1,2,4,5-tetrasubstituted imidazole derivatives **3**.

The reported microwave-assisted, catalyst- and solvent-free synthesis of 1,2,4,5-tetrasubstituted imidazoles **3** represents a rapid and efficient approach (Scheme 2 and Scheme 3). However, the claim of its environmental friendliness should be considered with caution, as the use of microwave radiation often requires specialized equipment, which may offset some of the advantages of green chemistry. Although the reaction is efficient and yields high isolated products (86–92%), this performance may be partly due to the limited substrate scope and optimized conditions rather than the inherent generality of the method. Furthermore, the absence of comparative data with traditional heating methods limits our ability to fully assess the energy and sustainability benefits of this approach. From a biological standpoint, the observed inhibition of EGFR^WT^ and EGFR^T790M^ activity is promising, but the study is preliminary in nature [53].

**Scheme 4:**
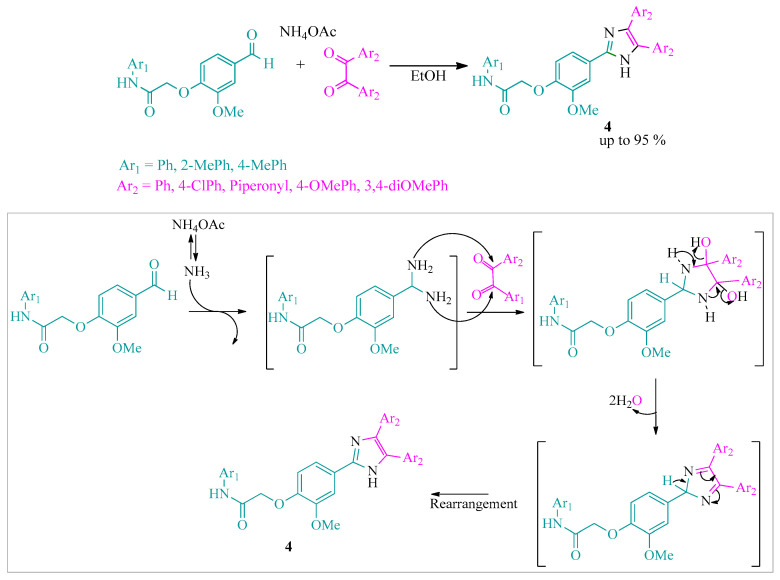
Three-component synthesis of biologically active imidazole derivatives **4** and the proposed reaction mechanism.

The antibacterial activity of 15 tri- and tetrasubstituted imidazole derivatives **4** synthesized via a catalyst-free protocol employing *O*-alkyl vanillins as aldehyde components were evaluated (Scheme 4). Although the catalyst-free approach offers synthetic simplicity, its practical significance is limited by the biological outcomes, as the majority of the obtained compounds exhibited antibacterial activity markedly lower than that of the reference drug ciprofloxacin. The observation that only a single derivative showed notable activity against *E. coli* suggests a weak or poorly optimized structure–activity relationship within this compound series. Consequently, the results indicate that incorporation of *O*-alkyl vanillin fragments alone is insufficient to achieve strong antibacterial potency and that further structural modification would be necessary to enhance biological performance [54].

**Scheme 5:**
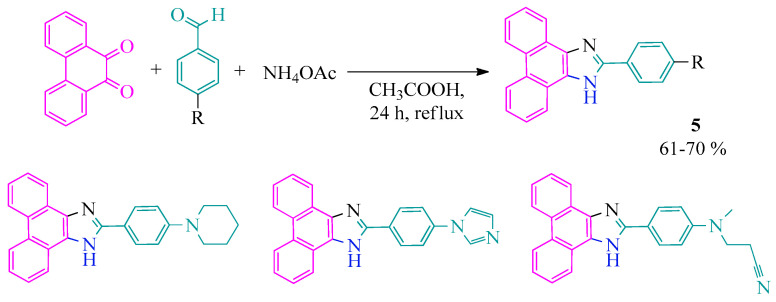
Synthesis of phenanthro[9,10–d]imidazole derivative **5**.

Phenanthro [9,10-d]imidazole derivatives **5** (see Scheme 5, 61–70%) were synthesized through condensation of the corresponding aldehyde (4-(piperidin-1-yl)benzaldehyde, 4-(1-imidazol-1-yl)benzaldehyde, 4-[(2-cyanoethyl)methylamino]benzaldehyde), 9,10-phenanthrenedione and ammonium acetate in acetic acid. This method is extremely simple and exhibits high tolerance to many functional groups. In addition, phenanthro [9,10-d]imidazole derivatives **5** precipitate after synthesis. The synthesized derivatives were characterized by high thermal stability (over 300 °C). Two of them (a and c) demonstrated a reversible oxidation process, which allowed for detailed spectroelectrochemical and electrochemical studies. The absorption and radiation properties were tested in four solvents of different polarities. These compounds showed high quantum yields ranging from 18% to 80% [55,56].

However, catalyst-free methods often require elevated temperatures or prolonged reaction times to achieve satisfactory conversions. These relatively harsh conditions may reduce functional group tolerance and limit the applicability of such protocols in complex molecule synthesis. In addition, catalyst-free approaches often show limited compatibility with aliphatic substrates, which generally possess lower intrinsic reactivity and may therefore require even more forcing conditions to undergo the desired transformation [57,58].

### 2.3. Organocatalysts and Bio-Based Catalysts

Organocatalysis has proven to be an effective strategy for promoting imidazole synthesis under mild conditions. A variety of organic acids and naturally derived catalysts, including ascorbic acid, glutamic acid, citric acid, and even fruit juices, have been successfully applied. These catalysts typically act by activating carbonyl groups and facilitating condensation steps in multicomponent reactions, thereby promoting the formation of the imidazole ring. Due to their biodegradability, low toxicity, and cost-effectiveness, naturally occurring organocatalysts represent attractive alternatives to traditional metal-based catalytic systems. In recent years, their application has gained increasing attention in sustainable heterocyclic synthesis, particularly in protocols designed to comply with green chemistry principles [59,60,61].

**Scheme 6:**
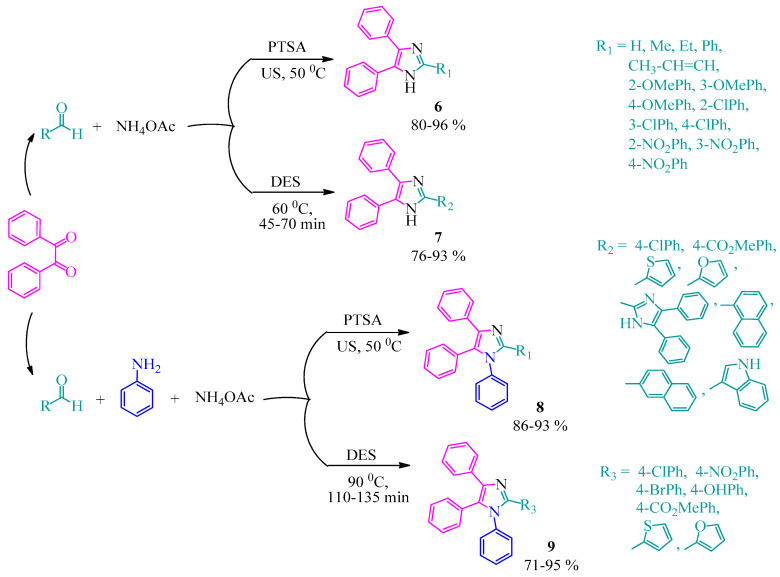
Synthesis of 2,4,5-tri- and 1,2,4,5-tetraphenyl imidazole derivatives using PTSA and DMU:SnCl_2_:HCl.

**Scheme 7:**
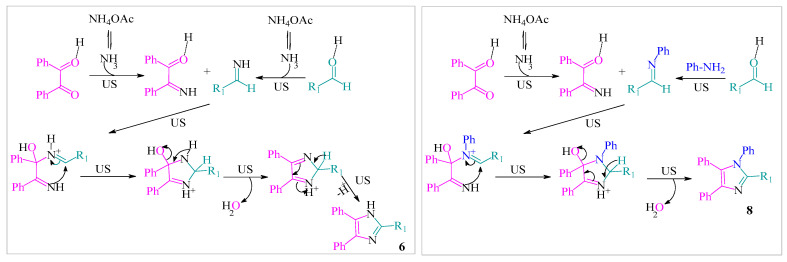
Mechanism of reaction of synthesis for tri- and tetrasubstituted imidazoles **6**, **8**.

The *p*-toluenesulfonic acid-catalyzed (PTSA) protocol for the synthesis of imidazole derivatives **6** and **8** demonstrates efficient access to structurally diverse substitution patterns (Scheme 6). Nevertheless, the reliance on relatively high catalyst loading (30 mol % PTSA) may limit the practical and economic attractiveness of the method. Although the optimized conditions (ethanol as solvent and ultrasonic irradiation) afford products in good yields (>80%), the improvement appears to be condition-dependent rather than indicative of an intrinsically robust or broadly generalizable transformation. In particular, the necessity of ultrasound assistance raises questions regarding scalability and reproducibility under standard synthetic conditions (Scheme 7) [62].

A protocol based on ternary deep eutectic solvent (DES) system composed of DMU:SnCl_2_:HCl was proposed for the preparation of tri- and tetrasubstituted imidazole derivatives **7**, **9** (Scheme 6). The study demonstrated that this DES system functions dually as both solvent and catalyst for the transformation. Fourteen trisubstituted derivatives **7** were obtained upon heating at 60 °C for 45–60 min, whereas the synthesis of 10 tetrasubstituted analogs **9** required heating at 90 °C for a slightly extended reaction time. The corresponding products were isolated in yields of 76–93% and 71–95%, respectively. Notably, the ternary DES system could be recycled and reused for up to five consecutive cycles without significant loss of catalytic activity. Despite these advantages, further investigation is required to confirm these findings [63].

**Scheme 8:**
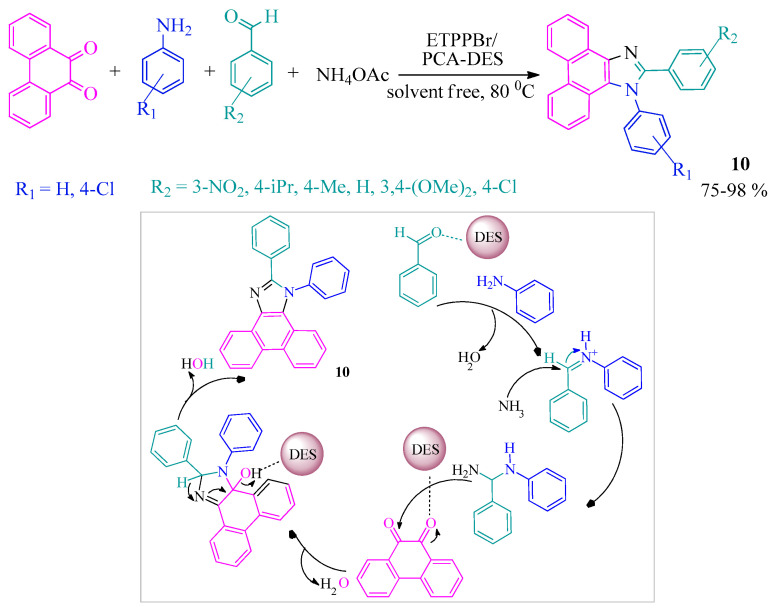
Synthesis of 1,2,4,5-tetrasubstituted imidazoles **10** by the DES catalyst and the proposed reaction mechanism.

The authors reported a method for the synthesis of a novel deep eutectic solvent based on a binary mixture of 3,4-dihydroxybenzoic acid and ethyltriphenylphosphonium bromide (ETPPBr/PCA-DES). The physicochemical properties of the prepared system were systematically investigated to evaluate its potential as a catalytic medium. To assess its catalytic performance, ETPPBr/PCA-DES was employed in the synthesis of 1,2,4,5-tetrasubstituted imidazoles **10**. The developed protocol enabled the efficient preparation of 12 structurally diverse imidazole derivatives **10** in excellent isolated yields (75–98%) within 20 min under solvent-free conditions. Notably, the transformation proceeded without the need for any additional organic solvent, highlighting the dual role of the DES as both reaction medium and catalyst. A plausible catalytic mechanism for the reaction was proposed (Scheme 8), providing insight into the activation pathway and the role of the DES components in promoting the transformation. However, the proposed reaction mechanism remains speculative due to the lack of experimental or computational validation. Overall, while the method demonstrates promising synthetic potential, further investigation is required to establish its practical advantages [64].

**Scheme 9:**
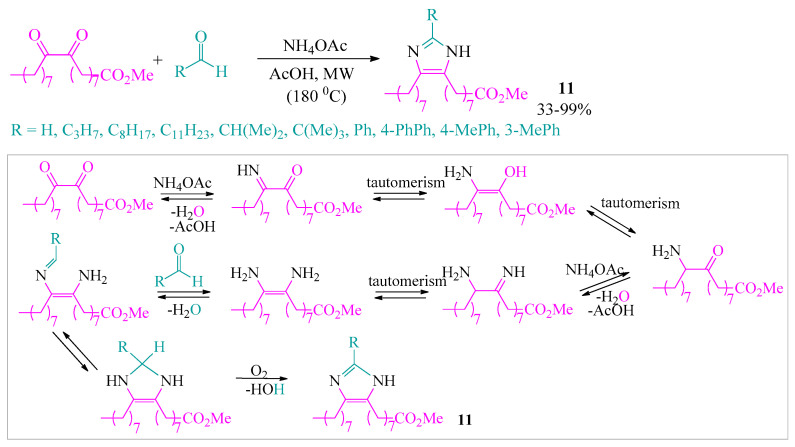
Synthetic route to fatty imidazoles and mechanistic proposal for the formation of fatty imidazole derivatives **11**.

The microwave-assisted synthesis of trisubstituted imidazole derivatives **11**, starting from methyl oleate, substituted benzaldehydes and ammonium acetate, using acetic acid as a catalyst, represents an efficient synthetic method (Scheme 9). However, the wide range of yields (33–99%) indicates significant variability depending on the substrate, suggesting that the method lacks consistency across different substitution patterns. Although high yields can be achieved in some cases, a plausible mechanism has been proposed to explain the observed reactivity and product formation [65].

**Scheme 10:**
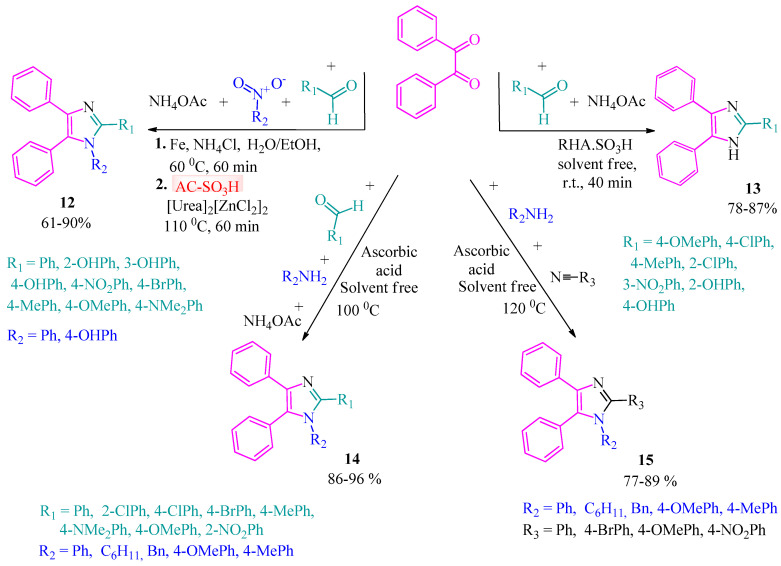
AC–SO_3_H, RHA–SO_3_H and ascorbic acid-catalyzed synthesis of polysubstituted imidazoles **12**–**15**.

**Scheme 11:**
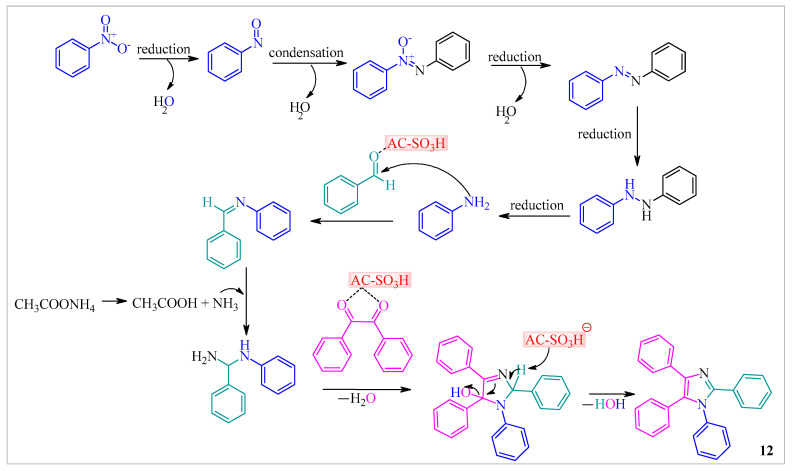
Proposed mechanism for the formation of polysubstituted imidazole frameworks **12** catalyzed by AC–SO_3_H.

The catalytic system based on a hybrid combination of sulfonic acid-functionalized amorphous carbon (AC–SO_3_H) and the deep eutectic mixture [Urea]_2_[ZnCl_2_]_2_ demonstrates an interesting concept of dual Brønsted–Lewis acid cooperation for the synthesis of tetrasubstituted imidazoles **12** (Scheme 10). However, despite the mechanistic appeal of the proposed “synergistic” activation, the study does not provide sufficient comparative data to confirm whether the observed activity truly exceeds that of the individual catalytic components, leaving the extent of synergy uncertain. Although the protocol affords the target imidazoles in good yields (average 73%) under mild conditions, the performance is not markedly superior to many simpler catalytic systems reported in the literature, which raises questions regarding its overall synthetic advantage. The reported recyclability of up to four cycles without significant loss of activity is a positive aspect; however, the relatively limited number of cycles does not fully establish long-term catalyst stability or industrial applicability. Furthermore, the proposed reaction mechanism, although plausible, remains speculative in the absence of direct experimental validation of the proposed cooperative acid activation pathway (Scheme 11) [66].

**Scheme 12:**
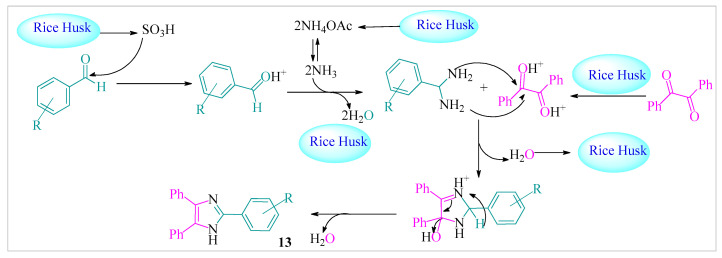
Proposed mechanism of RHA–SO_3_H-catalyzed synthesis of substituted imidazoles **13**.

In an effort to develop novel catalysts derived from renewable resources, rice husk ash was treated with sulfuric acid to afford a sulfonated material (RHA–SO_3_H). The catalytic performance of RHA–SO_3_H was subsequently evaluated in the synthesis of trisubstituted imidazoles **13** (Scheme 10). The results demonstrate that, under solvent-free conditions at ambient temperature, the target imidazole derivatives were obtained in good to excellent yields, reaching up to 87%. Nevertheless, the practical significance of this approach remains unclear. Thus, although a plausible reaction mechanism was proposed, the absence of comparative studies with previously reported systems makes it difficult to evaluate its relative efficiency (Scheme 12) [67].

**Scheme 13:**
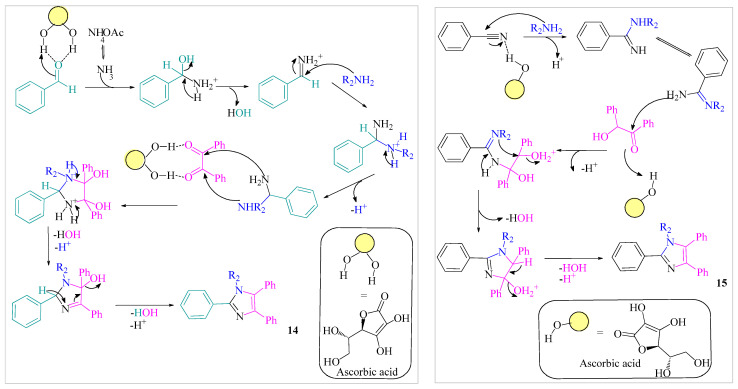
Plausible mechanisms of ascorbic acid-catalyzed synthesis of different 1,2,4,5-tetrasubstituted imidazoles **14**, **15**.

The authors developed a method for the synthesis of 1,2,4,5-tetrasubstituted imidazoles using vitamin C (ascorbic acid) as a catalyzing agent. For that reason, two model reactions were employed. The first one is a classic four-component reaction yielding 1,2,4,5-tetrasubstituted imidazoles **14**, at 100 °C, while the other is a three-component reaction of benzoin, amine, and nitrile compounds at 120 °C yielding same imidazole scaffold **15**, (Scheme 10). Both reactions showed good catalytic potential of ascorbic acid in solvent-free conditions with excellent yields of target products, ranging from 86% to 96% for the first reaction and from 77% to 89% for the second reaction. Moreover, plausible reaction mechanisms were proposed to better understand the catalytic effect of vitamin C and compared the efficiency of the present catalyst with already reported catalytic systems (Scheme 13) [68].

**Scheme 14:**
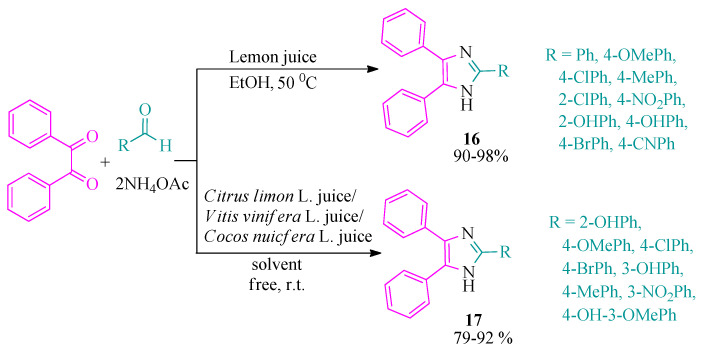
Synthesis of 2,4,5-triaryl–1*H*–imidazole **16**, **17**.

Despite the fact that use of naturally derived catalysts has attracted increasing attention in the context of green and cost-effective synthesis of imidazole derivatives **16**, the application of lemon juice as a catalytic medium raises questions regarding its definition as a truly catalytic system, given its poorly defined composition and variability in organic acid content (Scheme 14). While the reported three-component reaction proceeds efficiently under mild conditions, the reliance on relatively large volumes of lemon juice (4–10 mL) challenges its classification as a catalytic amount in a strict mechanistic sense and may instead suggest a solvent-assisted or acid-mediated process rather than true catalysis. Even though high yields (>90%) are achieved within short reaction times, the absence of control experiments (e.g., using defined concentrations of citric acid or pH-matched systems) limits the ability to attribute the observed efficiency specifically to the complex natural matrix of lemon juice. Consequently, the claimed advantage of using a biogenic catalyst remains partially unsubstantiated, as similar performance might be attainable with simple, well-defined organic acids under comparable conditions. Thus, while the study aligns with green chemistry principles in concept, the mechanistic ambiguity and lack of quantitative benchmarking weaken the strength of its catalytic claim [69].

**Scheme 15:**
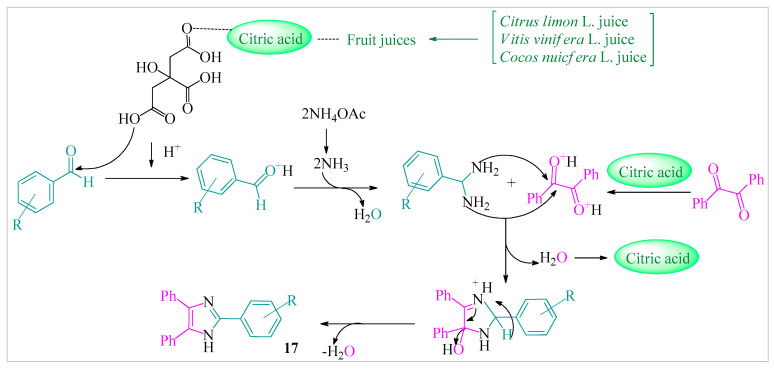
Fruit juice-catalyzed synthesis of substituted imidazoles and proposed mechanism.

Although the exploration of fruit-juice-based biogenic catalytic systems, such as those derived from *Citrus limon*, *Vitis vinifera*, and *Cocos nucifera*, aligns with the principles of green and sustainable chemistry, there are important questions regarding the validity and reproducibility of these undefined catalytic media. While all three of these systems are reported to yield 2,4,5-trisubstituted imidazole derivatives **17** in good yields (greater than 80%) (see Scheme 14 and Scheme 15), the lack of standardized compositional analysis of the juices limits the reproducibility and generality of the reported findings. A plausible mechanistic pathway has been proposed, but it remains uncertain due to the complexity and variability of the catalytic systems. Overall, while the procedure is straightforward and yields are promising, the mechanistic explanation and novelty of the process remain insufficiently supported [70].

**Scheme 16:**
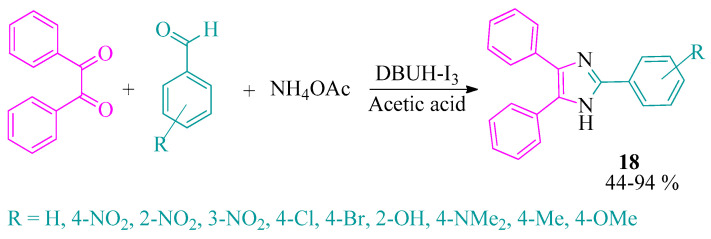
DBUH-I_3_ catalyzed synthesis of divergent 2,4,5-trisubstituted imidazoles **18**.

The 3,5,6,7,8,9-hexahydro-2*H*-imidazo[1,2-a]azepin-1-ium-iodine-iodide (DBUH-I_3_), was developed and utilized in the synthesis of 2,4,5-trisubstituted imidazole compounds **18** in a recent research paper. Based on the method of synthesis presented by the researchers, numerous imidazoles **18** can be produced in good yields (maximum yield reported in the source was 94%), utilizing readily available reagents such as ammonium acetate, substituted aromatic aldehydes, and 1,2-diketones (Scheme 16). In their work, the authors note that aliphatic aldehydes are not appropriate for this method of synthesizing imidazole derivatives **18**. Nevertheless, the practical significance of this approach remains unclear [71].

**Scheme 17:**
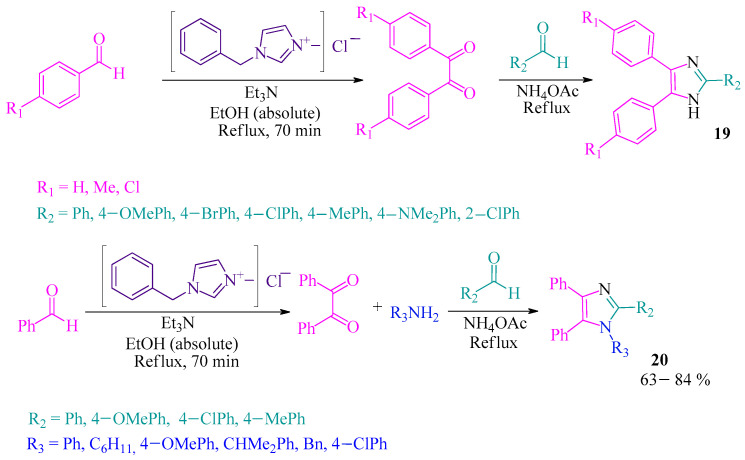
Synthesis of 2,4,5-tri- and 1,2,4,5-tetrasubstituted imidazoles **19**, **20**.

The reported strategy employing imidazole-derived precatalysts for the synthesis of tri- and tetrasubstituted imidazole derivatives **19** and **20** involves a sequential two-step process based on the in situ generation of 1,2-diketones through *N*-heterocyclic carbene (NHC) catalysis (see Scheme 17). This approach highlights the synthetic utility of NHC-mediated aldehyde coupling reactions as a route to key diketone intermediates, which subsequently undergo condensation with aldehydes and ammonium acetate to afford the target imidazole frameworks. While the methodology provides access to a series of 20 compounds **19**, **20** with good yields (up to 90%), the overall efficiency of the protocol may be limited by the multistep nature of the process, which potentially reduces operational simplicity compared to direct multicomponent imidazole syntheses. Moreover, the study does not clearly establish whether the use of imidazole-derived precatalysts offers a significant advantage over previously reported NHC systems or conventional catalytic approaches for diketone formation. In addition, the substrate scope and functional group tolerance appear insufficiently explored, which restricts evaluation of the general applicability of the method. Consequently, although the strategy demonstrates the versatility of NHC catalysis in constructing imidazole precursors, further comparative and mechanistic studies would be required to fully assess its synthetic value [72].

**Scheme 18:**
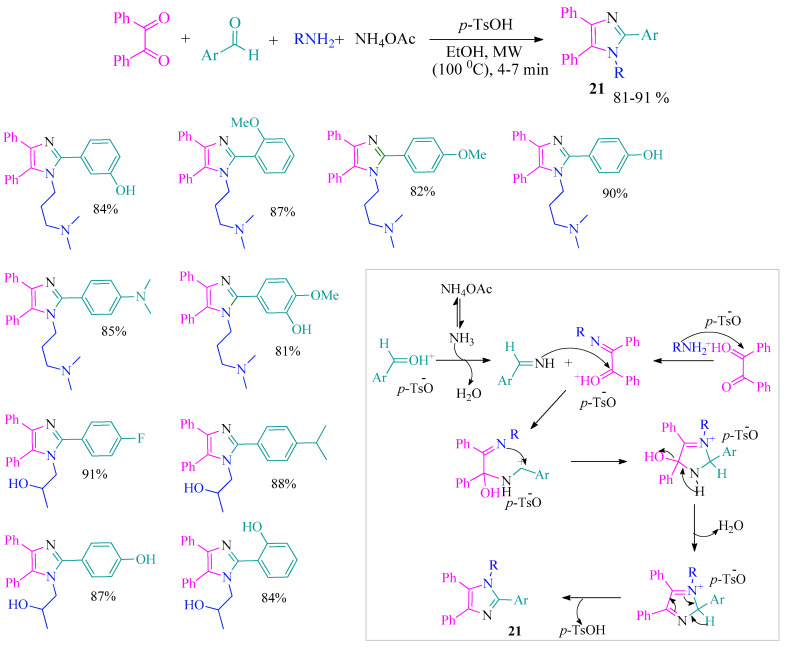
Synthesis of polysubstituted imidazoles and proposed mechanism for the formation.

In spite of the fact that the use of para-toluenesulfonic acid (*p*-TsOH) as a Brønsted acid catalyst enables the efficient synthesis of polysubstituted imidazole derivatives **21** via a four-component reaction, the methodological novelty appears limited given the widespread use of *p*-TsOH in similar acid-catalyzed multicomponent transformations (Scheme 18). While high yields (up to 91%) were obtained, the study does not clearly demonstrate whether these outcomes represent a significant improvement over existing catalytic systems or are simply consistent with established acid-promoted protocols. Moreover, although two different amino derivatives were employed, the extent of substrate generality remains insufficiently explored, which limits assessment of the true versatility of the method. Overall, despite the operational simplicity and acceptable yields, the synthetic advantage and mechanistic insight provided by the study are relatively modest in the context of previously reported imidazole syntheses [73].

**Scheme 19:**
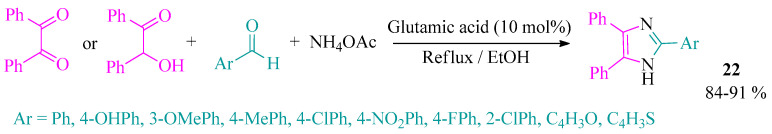
Synthesis of 2,4,5-triaryl–1*H*–imidazoles using glutamic acid as catalyst.

The authors investigated a method for the synthesis of trisubstituted imidazoles **22** through a multicomponent reaction under mild conditions, using glutamic acid as a catalyst and ethanol as a solvent (Scheme 19). This method allowed the authors to obtain 12 different compounds **22** with yields up to 91%. However, the absence of comparative studies with previously reported systems makes it difficult to evaluate its relative efficiency. Therefore, while the protocol presents an interesting approach, further research is required to validate its reliability and underlying mechanisms [74].

These systems are particularly attractive due to their low cost, biodegradability, and compatibility with benign solvents such as ethanol. Furthermore, their origin from renewable resources and low toxicity make them suitable for sustainable synthetic applications. The use of such systems can significantly reduce environmental impact by decreasing the need for hazardous solvents and energy-intensive conditions. In many cases, they also enable straightforward product isolation, thereby improving the overall efficiency of the synthetic process. Table 1 provides a summary of representative other examples of organic and biological catalytic systems reported for the synthesis of imidazole derivatives. These catalytic systems include organic acids, amino acids, catalysts derived from natural products, all of which have been successfully utilized in multicomponent reactions under mild and environmentally friendly conditions. In many cases, these systems offer advantages such as low toxicity, ease of operation, and compatibility with environmentally friendly solvents.

### 2.4. Metal-Based Catalysts

Transition metal catalysis has greatly expanded the range of the classical Debus–Radziszewski reaction, allowing for improved control over reactivity and compatibility with substrates. Catalysts based on copper, iron, manganese, titanium, and lanthanide salts have been particularly effective in facilitating cyclocondensation reactions by activating carbonyl intermediates and stabilizing key reaction pathways. Compared to catalyst-free methods, metal-catalyzed reactions typically exhibit higher reaction rates, enhanced selectivity, and greater tolerance towards functionalized aldehydes and diketones. However, the use of transition metals can introduce challenges related to catalyst recovery and potential metal contamination, particularly in pharmaceutical synthesis. As a result, recent research has focused increasingly on developing recyclable, heterogeneous catalysts and metal-free alternatives that retain the high efficiency of metal-based catalyzed systems, while minimizing their environmental impact [80,81,82].

**Scheme 20:**
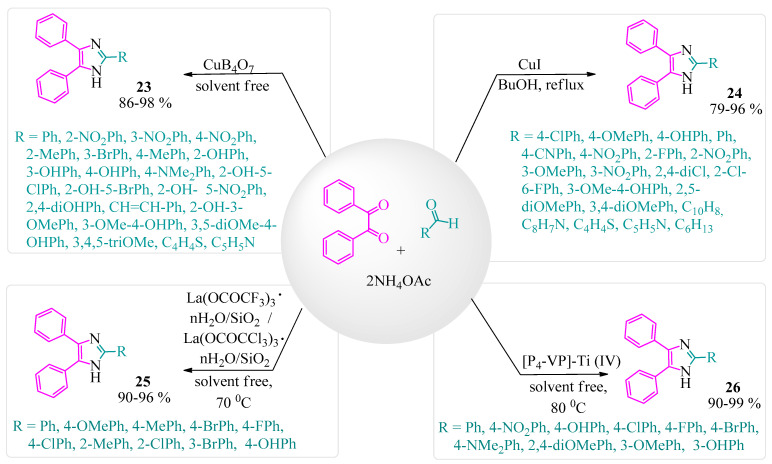
Synthesis of 2,4,5-triarylimidazole using different metal catalysts.

**Scheme 21:**
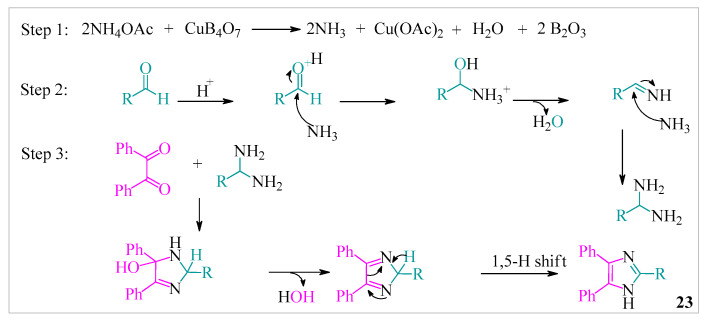
The proposed mechanism of the synthesis of 2,4,5-triarylimidazole using CuB_4_O_7_.

Although copper borate (CuB_4_O_7_) is presented as a simple catalytic system for the synthesis of 2,4,5-trisubstituted imidazole derivatives **23** (as shown in Scheme 20), the claim of environmental advantage remains only partially substantiated, as the study does not provide a quantitative assessment of its green chemistry metrics (e.g., E-factor, energy consumption, or catalyst recovery efficiency). While the reported synthesis of 26 derivatives **23** with high yields (86–98%) suggests good synthetic efficiency, such consistently high yields may reflect a limited structural complexity of the tested substrates rather than a broadly robust catalytic performance. In addition, the absence of a solvent is emphasized as a key advantage, and the paper provides comparisons with solvent-based catalytic systems and alternative catalytic systems, which improves the assessment of the actual practical effect (Scheme 21) [83].

**Scheme 22:**
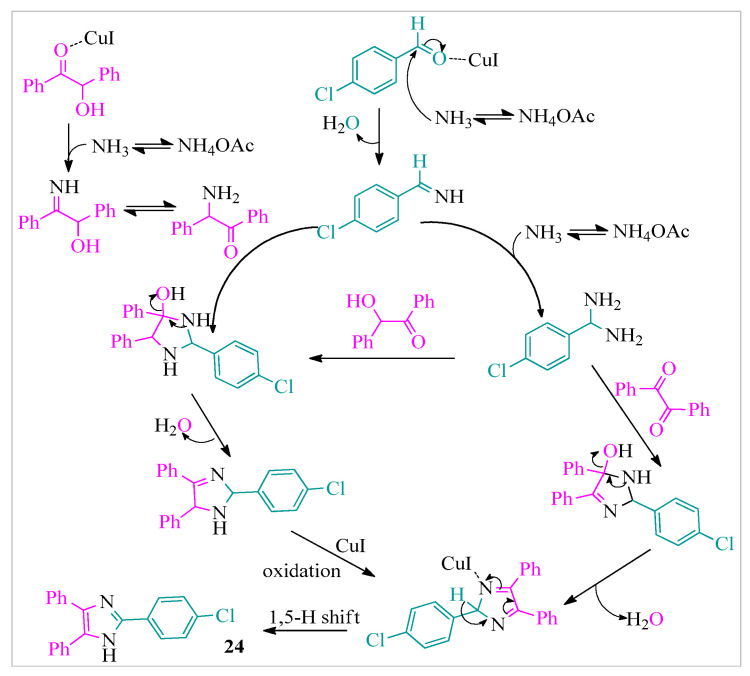
Copper catalyzed multicomponent reaction and plausible reaction mechanism.

The authors reported that a relatively simple and practical approach for the synthesis of 2,4,5-trisubstituted imidazole derivatives **24** was investigated using monovalent copper as a catalyst. The procedure provides efficient access to obtain 40 different imidazole products **24** with yields up to 96% using CuI and butanol as the solvent (Scheme 20 and Scheme 22). Despite these advantages, there are certain aspects of the methodology that require further evaluation. The lack of comparative studies with previous systems makes it challenging to assess its effectiveness [84].

**Scheme 23:**
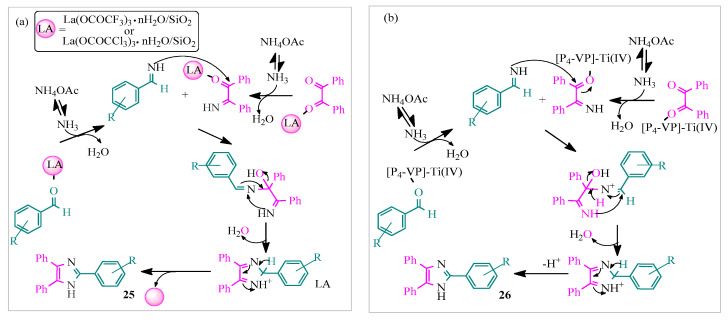
Proposed mechanisms for (**a**) the silica-supported lanthanum and (**b**) titanium-catalyzed synthesis of 2,4,5-triarylimidazoles **25**, **26**.

A protocol based on lanthanum trifluoride and trichloride supported on silica (La(OCOCF_3_)_3_∙nH_2_O/SiO_2_ and La(OCOCCl_3_)_3_∙nH_2_O/SiO_2_ was proposed for the preparation of 2,4,5-trisubstituted imidazole compounds **25**. These catalysts enable the production of imidazole derivatives **25** with an excellent yield of up to 96% in a short time period without the use of 20 and solvents (Scheme 23). Furthermore, these catalysts offer benefits, including their non-toxic nature and the retention of good catalytic activity even after multiple uses [85].

A titanium tetrachloride-based catalytic system cross-linked with poly(4-vinylpyridine) has been reported for the selective synthesis of 2,4,5-trisubstituted imidazole derivatives **26**. The method enables the formation of 2-aryl-4,5-diphenyl imidazoles under solvent-free conditions at temperatures (80 °C), affording high yields of the desired products (up to 99%). While the solvent-free nature of the protocol and the reported efficiency suggest potential practical advantages, the use of titanium tetrachloride—a highly moisture-sensitive and corrosive reagent—raises concerns regarding operational safety and environmental compatibility, which may partially offset the claimed sustainability benefits (Scheme 23) [86].

**Scheme 24:**
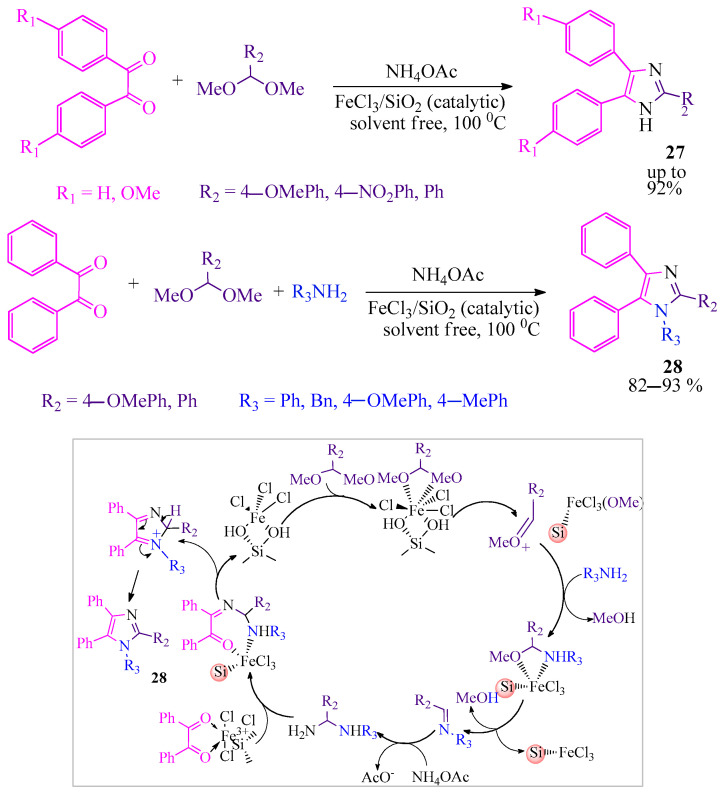
The formation of 2,4,5-triphenylimidazole **27**, **28**.

The authors developed a modified method for the synthesis of polysubstituted imidazole derivatives **27**, **28** using acetals and benzyl diketones with ammonium acetate or amines as a nitrogen source, and FeCl_3_/SiO_2_ as a catalyst (as shown in Scheme 24). The reaction yields excellent results, with up to 92% yield, without the need for a solvent, at 100 °C, and in a relatively short period of time. The reported catalytic performance appears promising; however, its superiority over existing systems remains unclear. Despite promising initial results, further optimization and mechanistic clarification are required to fully establish the catalytic system [87].

**Scheme 25:**
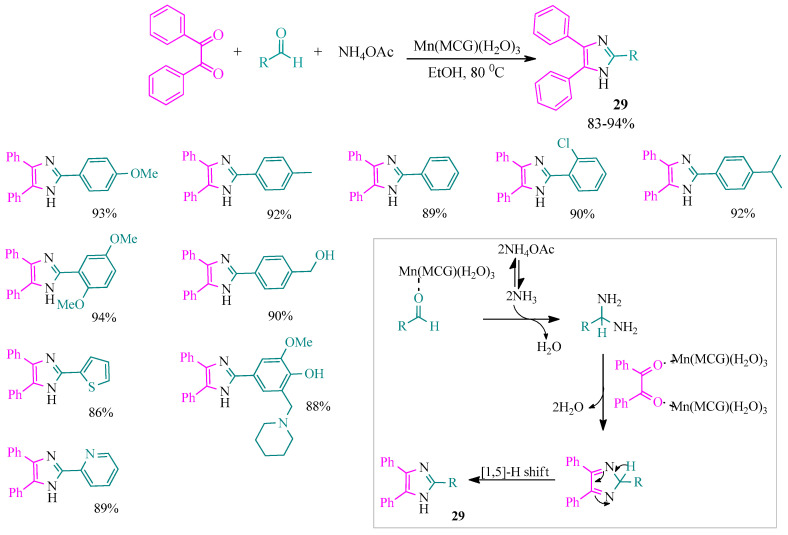
The Debus–Radziszewski imidazole synthesis catalyzed with Mn(MCG)(H_2_O)_3_ particles.

A manganese complex based on [7-hydroxy-4-methyl-8-coumarinyl]glycine ([Mn(MCG)(H_2_O_3_]) has been proposed as a catalyst for the synthesis of 2,4,5-trisubstituted imidazole derivatives **29**, as shown in Scheme 25. The protocol enables the formation of the target compounds in ethanol at 80 °C, affording sixteen derivatives with high yields (83–94%). These results suggest that the catalyst is capable of efficiently promoting the transformation under moderate conditions. In particular, the preparation of a specialized metal–organic complex may introduce additional synthetic steps and costs compared with simpler catalytic systems commonly used for imidazole synthesis. The authors reported that [Mn(MCG)(H_2_O)_3_] can be used as a heterogeneous catalyst in various organic reactions. Consequently, the catalyst demonstrates promising activity in the reported reactions [88].

Despite their advantages, issues related to metal contamination and catalyst recovery may limit their applicability in pharmaceutical contexts. Trace amounts of transition metals originating from catalytic systems can remain in the final reaction mixture and may contaminate active pharmaceutical ingredients (APIs) because such metal residues provide no therapeutic benefit and may pose toxicological risks. In addition, catalytic reactions are often performed in late stages of pharmaceutical synthesis, which increases the risk of residual metal impurities persist in the final product. Therefore, additional purification steps—such as chromatography, metal scavengers, or adsorption techniques—are frequently required to reduce metal concentrations to acceptable limits. These extra purification procedures may increase production costs and reduce overall process efficiency, particularly in large-scale manufacturing. Furthermore, the recovery and recycling of homogeneous metal catalysts can be technically challenging, which further complicates their practical application in pharmaceutical process chemistry. For these reasons, the development of metal-free or organocatalytic alternatives has received increasing attention in recent years as a strategy to minimize contamination risks and improve the sustainability of synthetic methodologies [89,90].

### 2.5. Nanocatalysts and Supported Systems

Nanostructured catalysts have emerged as particularly attractive systems for imidazole synthesis due to their high surface-to-volume ratio and tunable physicochemical properties. Magnetic nanoparticles, metal oxide nanomaterials, and carbon-based catalysts have demonstrated remarkable catalytic efficiency in multicomponent imidazole synthesis, frequently enabling high yields under mild reaction conditions. In addition to improved catalytic activity, these materials offer practical advantages such as facile recovery through magnetic separation or filtration and the possibility of multiple reuse cycles. However, the preparation of well-defined nanocatalysts and the prevention of nanoparticle aggregation remain important challenges that may influence catalytic performance. Consequently, current research is increasingly directed towards the development of stable, functionalized nanomaterials capable of maintaining high activity over extended recycling cycles [91,92].

**Scheme 26:**
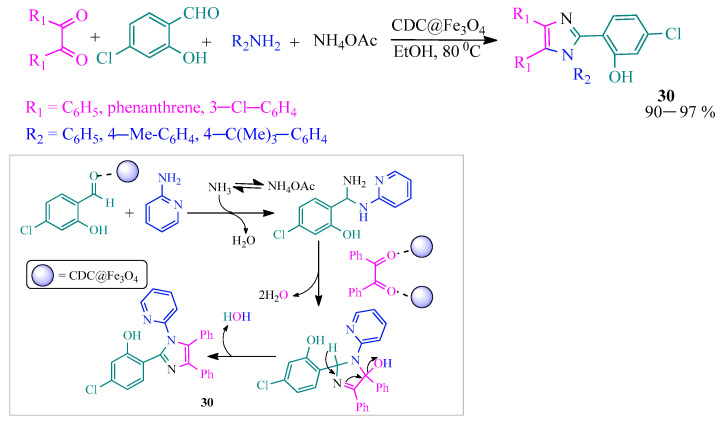
Magnetic chlorodeoxycellulose for imidazole derivative **30** catalytic synthesis.

A magnetic chlorodeoxycellulose/ferroferric oxide composite (CDC@Fe_3_O_4_) has been reported as a heterogeneous photocatalyst for the synthesis of tetrasubstituted imidazole derivatives **30** (Scheme 26). The protocol enables the formation of the desired products in ethanol under ultrasound irradiation at room temperature, affording nine derivatives within 30 min in high yields (90–97%) while employing a low catalyst loading (5 mg). Although these results suggest efficient catalytic performance, the practical advantages of the method require further clarification. In particular, the simultaneous reliance on ultrasound irradiation and photocatalytic activation complicates evaluation of the true catalytic contribution of the CDC@Fe_3_O_4_ material. Furthermore, while the catalyst can reportedly be recovered magnetically and reused for up to five cycles, such recyclability studies remain relatively limited and do not fully establish long-term catalyst stability or structural integrity. The substrate scope also appears modest, as only a small number of imidazole derivatives were examined. Consequently, although the magnetic catalyst represents an interesting approach combining heterogeneous catalysis with convenient magnetic separation, additional studies would be necessary to confirm its broader applicability, mechanistic role, and comparative advantages over simpler catalytic systems [93].

**Scheme 27:**
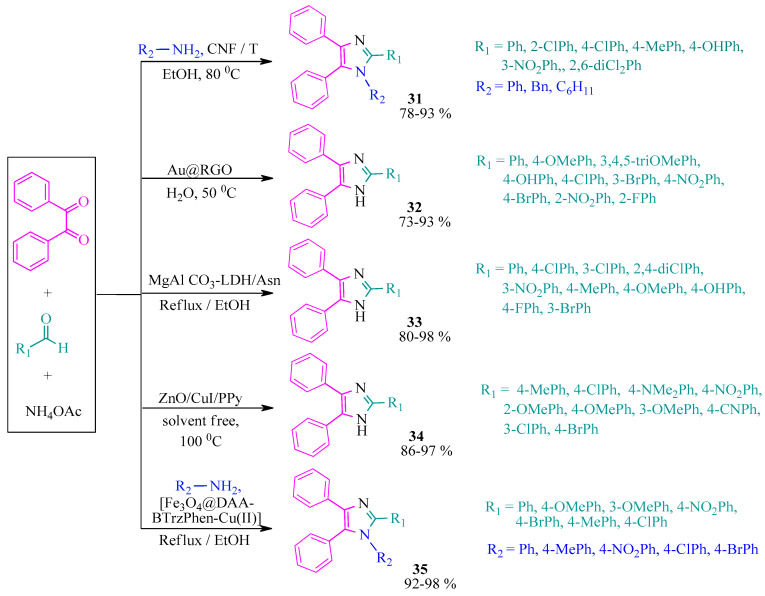
Nano-catalyzed synthesis of tri- and tetrasubstituted imidazoles **31**–**35**.

**Scheme 28:**
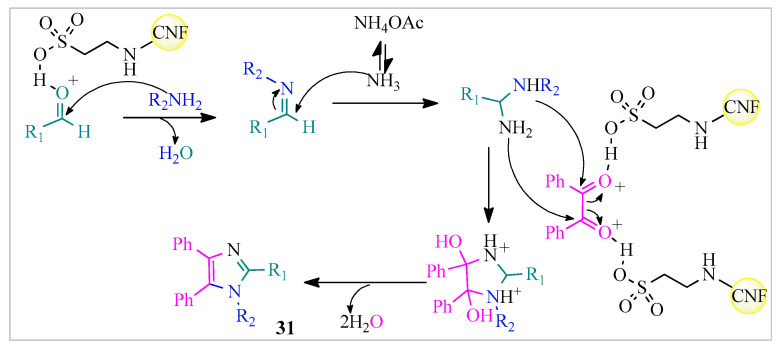
Probable mechanism of the CNF/T-catalyzed the synthesis of substituted imidazoles **31**.

An acid carbon nanofiber modified with taurine catalytic system (CNF/T) was developed as a Brønsted acid catalyst for the synthesis of 1,2,4,5-tetrasubstituted imidazole derivatives **31** (Scheme 27 and Scheme 28). It was shown that the optimum conditions for using this catalytic system are 0.03 g of CNF/T, ethanol as the solvent, and temperature of 80 °C. In such conditions it is easy to achieve 78–93% yields. Although catalyst recyclability was demonstrated, the limited number of cycles does not fully establish long-term stability. Therefore, further studies are required to establish its general applicability [94].

A gold nanocatalyst supported on graphene oxide (Au@RGO) has been shown to be effective in the production of 2,4,5-trisubstituted imidazole derivatives **32** with high yields, up to 93% (Scheme 27). This catalyst has several advantages, including ease of use, the utilization of water as a green solvent, and the potential for reuse up to seven times without significant loss of activity and provides a significant improvement compared to previously reported methodologies [95].

**Scheme 29:**
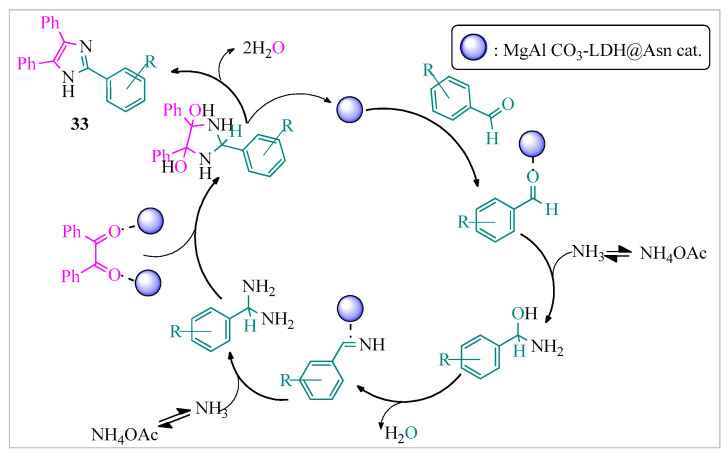
Possible reaction mechanism for the synthesis of trisubstituted derivatives of 2,4,5-(1*H*)-imidazoles catalyzed with MgAl CO_3_-LDH@Asn biocatalyst.

A nanocomposite catalyst based on a magnesium aluminum carbonate layered double hydroxide (MgAlCO_3_-LDH@Asn) has been reported for the synthesis of 2,4,5-trisubstituted imidazole derivatives **33** (Scheme 27 and Scheme 29). The system enables the formation of the target compounds in ethanol using a small catalyst loading (20 mg), affording 12 products in high yields (80–98%). While these results indicate that the LDH-based material can efficiently promote the transformation under mild conditions, the practical advantages of the catalyst remain to be fully clarified. In particular, layered double hydroxides have been widely investigated as heterogeneous catalysts, and the study does not clearly demonstrate whether the asparagine-modified LDH provides a substantial improvement over previously reported LDH systems. Furthermore, although the recyclability of the catalyst was demonstrated for up to four cycles without a significant decrease in activity, a limited number of reusability experiments does not definitively establish the long-term stability of the catalyst. The relatively small number of substrates tested also limits the evaluation of the overall applicability of the method. Overall, while the LDH-based nanocomposite presents an interesting heterogeneous catalytic system for imidazole synthesis, additional mechanistic research would be required to confirm its superiority over existing catalytic materials [96].

**Scheme 30:**
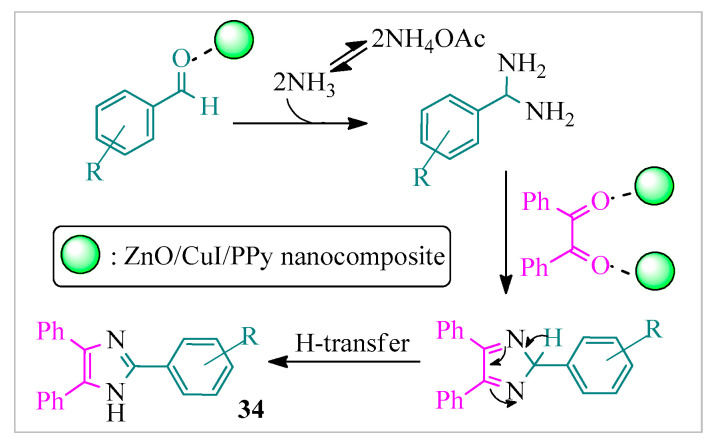
Proposed mechanism for optimization the synthesis of 2,4,5-trisubstituted imidazoles **34** by ZnO/CuI/PPy nanocatalyst.

Recently, an environmentally friendly nanocomposite system consisting of zinc oxide (ZnO), copper(I) iodide (CuI), and polypyrrole (PPy) was developed for the preparation of synthesis of 2,4,5-trisubstituted imidazole derivatives **34** (Scheme 27). This approach provides efficient access to obtain 12 compounds **34** in high yields (>85%) on the solvent-free system. The catalytic mechanism appears to involve activation of the carbonyl group of aldehydes, which react with ammonium acetate to generate an intermediate diamine. This diamine then reacts with a dicarbonyl compound to produce an additional intermediate that, upon proton transfer, transforms into a substituted imidazole product (see Scheme 30). Additionally, the authors of this study claim that the catalyst maintains a substantial portion of its efficacy even after six cycles of use [97].

**Scheme 31:**
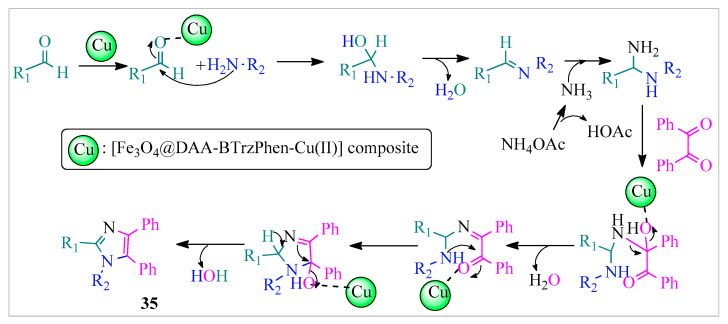
Proposed reaction mechanism of the synthesis of tetrasubstituted imidazoles **35** over the catalysis of the [Fe_3_O_4_@DAA-BTrzPhen–Cu(II)] composite.

A nanomagnetic copper(II) complex based on a bistriazolylphenanthroline ligand immobilized on Fe_3_O_4_ (Fe_3_O_4_@DAA-BTrzPhen-Cu(II)) has been reported as a heterogeneous catalyst for the synthesis of tetrasubstituted imidazole derivatives **35** via a multicomponent condensation of aldehydes, 1,2-diketones, aniline derivatives, and ammonium acetate (Scheme 27). The protocol affords the desired products in excellent yields (92–98%) under relatively mild conditions in ethanol, and the catalyst can reportedly be recovered magnetically and reused for up to seven cycles. However, the practical advantages of this system remain somewhat uncertain, as the preparation of a structurally complex nanomagnetic catalyst may involve multiple synthetic steps compared with simpler catalytic systems commonly employed for imidazole synthesis. Furthermore, although the reported substrate scope includes 23 examples, the study does not provide detailed benchmarking against established catalytic protocols, making it difficult to determine whether the observed performance represents a substantial improvement. While catalyst recyclability up to seven cycles is encouraging, additional studies would be necessary to confirm long-term structural stability and catalytic durability. In addition, the proposed reaction mechanism remains hypothetical in the absence of computational evidence supporting the catalytic pathway (Scheme 31). Overall, while the nanomagnetic Cu(II) complex represents an interesting design combining coordination catalysis with magnetic recoverability, further mechanistic investigations would be required to fully establish its practical advantages [98].

**Scheme 32:**
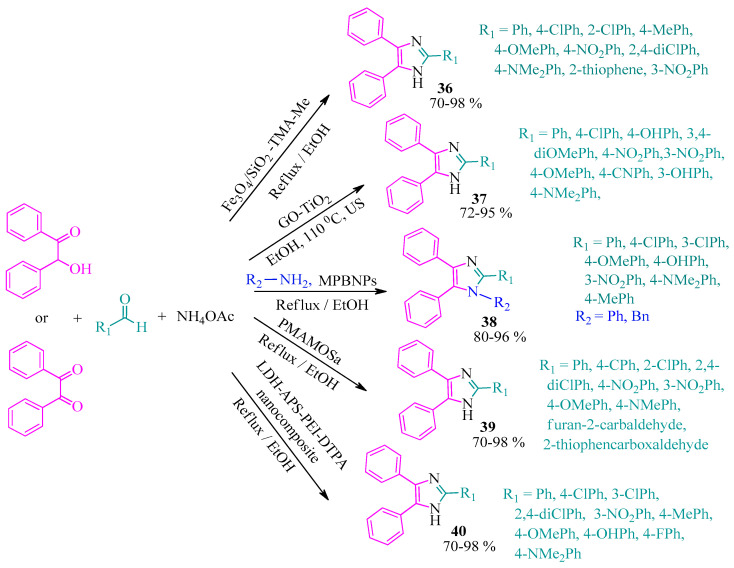
Synthesis of substituted imidazoles **36**–**40** using different nanoparticles.

**Scheme 33:**
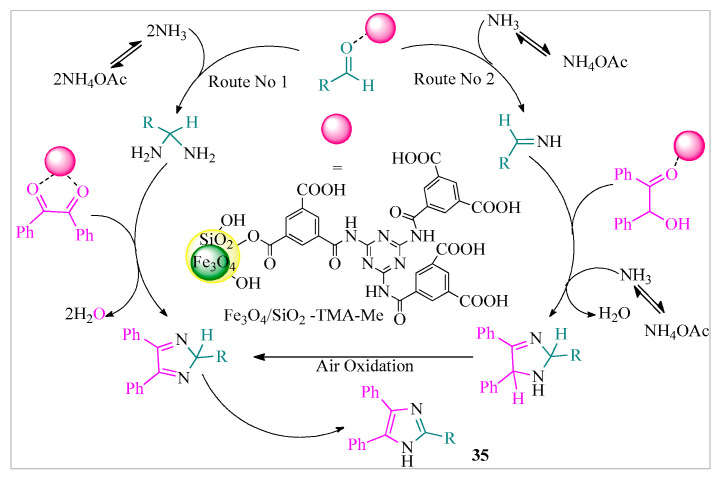
Proposed mechanism for Fe_3_O_4_/SiO_2_-TMA-Me nanocomposite for the three-component condensation of benzil or benzoin, aldehydes and ammonium acetate to afford imidazole derivative **36**.

The pseudo-supramolecular Fe_3_O_4_/SiO_2_ decorated trimesic acid–melamine (Fe_3_O_4_/SiO_2_-TMA-Me) nanocomposite was successfully synthesized and tested for catalytic properties for obtaining 2,4,5-trisubstituted imidazole products **36**. For such purposes, a three-component condensation of benzoin or benzil, various benzaldehydes, and ammonia acetate was chosen to afford respective imidazoles **36** (Scheme 32 and Scheme 33). Low catalyst loading, good to excellent yields reaching 98%, easy isolation of the magnetic catalyst from the reaction mixture, and its capability to be reused up to seven times without significant loss of its catalytic activity constitute the main merits of this synthesis protocol [99].

**Scheme 34:**
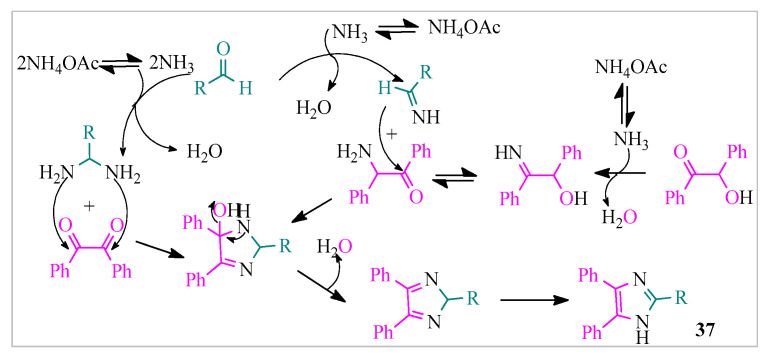
Potential mechanism of catalytic action of GO-TiO_2_ for the synthesis of 2,4,5-trisubstituted imidazoles **37**.

In their recent research, a team of scientists has synthesized a non-toxic and under-studied nanocatalyst based on titanium dioxide supported on graphene oxide (GO-TiO_2_), and used it to produce a range of 2,4,5-trisubstituted imidazole derivatives **37** (Scheme 32). In addition to determining the optimal synthesis conditions, the researchers also compared two approaches for producing imidazoles **37**: a conventional method and an ultrasonic-assisted method. Based on results from 20 experimental runs, it was found that ultrasonic irradiation yields slightly higher product yields (80–93%) compared to the conventional approach (72–95%), although the latter can also be used to consistently produce imidazole derivatives with stability. The research team has also analyzed the potential mechanism of catalytic action of GO-TiO_2_ (as shown in Scheme 34) [100].

**Scheme 35:**
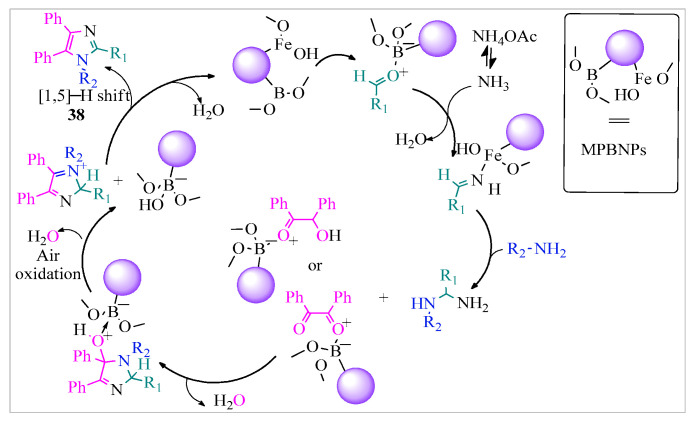
Plausible reaction mechanism of one-pot four-component synthesis of tetrasubstituted imidazoles **38** catalyzed by the magnetic polyborate nanoparticles (MPBNPs).

The synthesis and characterization of magnetic polyborate nanoparticles (MPBNPs) were reported in a recent study. The authors demonstrated that these nanoparticles can efficiently catalyze a four-component condensation reaction, leading to the formation of various 1,2,4,5-tetrasubstituted imidazole derivatives **38**, as shown in Scheme 32. Under MPBNP catalysis, the reaction proceeds under mild conditions and affords excellent yields of up to 96%. Additionally, a plausible reaction mechanism has been proposed (Scheme 35). From a critical perspective, although the reported catalytic performance is promising, several aspects require further clarification. First, the study would benefit from a more comprehensive comparison with existing catalytic systems to better establish the relative advantage of MPBNPs in terms of efficiency, cost, and environmental impact. Second, the reusability and long-term stability of the nanoparticles should be more rigorously evaluated, as magnetic nanocatalysts are often prone to aggregation or gradual deactivation over multiple cycles. Third, mechanistic proposals remain largely speculative unless supported by additional kinetic studies or spectroscopic evidence of intermediates. Therefore, while the reported system appears effective under the described conditions, further validation is necessary to confirm its practical applicability and mechanistic robustness [101].

**Scheme 36:**
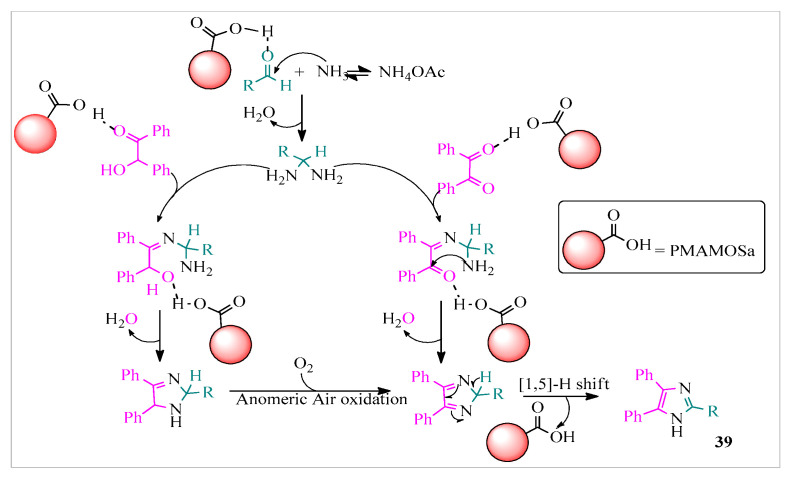
Proposed mechanism for PMAMOS-catalyzed synthesis of imidazole derivatives **39** through a multicomponent reaction of benzil or benzoin aldehyde derivatives, and ammonium acetate in EtOH under reflux conditions.

Recently, nanospheres based on a mesoporous organosilicate that was cross-linked with pyromellitic diacid (PMAMOS) was developed for the preparation of trisubstituted imidazoles **39**. The method produces the desired products with high yields, reaching up to 98%, under mild conditions and in a short time period (Scheme 32 and Scheme 36). They also found that the nanospheres could be reused up to five times without significant loss of efficiency, measured the acidity of PMAMOS using the back titration method and made comparison with other catalysts [102].

**Scheme 37:**
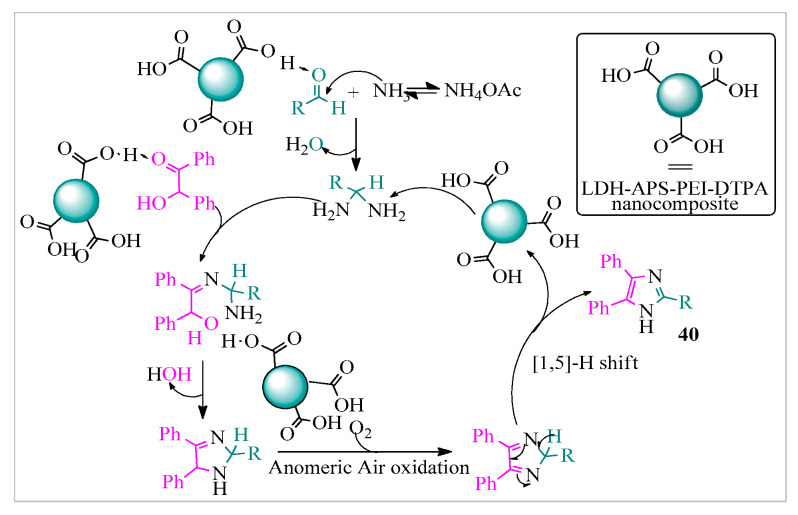
Proposed mechanism for LDH/polymer composite-catalyzed synthesis of imidazole derivatives **40**.

A novel nanocomposite material based on a layered double hydroxide polymer was synthesized for obtaining 11 different trisubstituted imidazole products **40** with good yields (70–98%) (see Scheme 32 and Scheme 37). The authors state that the use of LDH-APS-PEI-DTPA in the synthesis of imidazoles **40** is accompanied by low cost, good yields, and ease of product recovery from the reaction mixture. Additionally, the nanocomposite can be reused up to six times without a 20% decrease in catalytic efficiency [103].

**Scheme 38:**
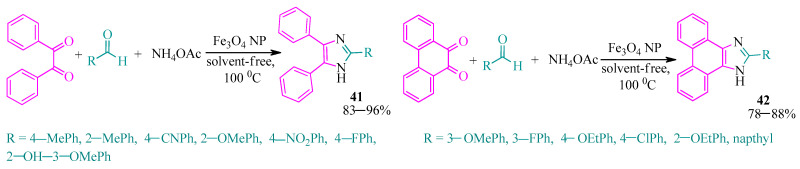
Fe_3_O_4_ NPs catalyzed the synthesis of tri-substituted imidazoles **41**, **42**.

The Fe_3_O_4_ nanoparticles modified with nitrogen-doped quantum dots (Fe_3_O_4_ NPs) were synthesized and subsequently evaluated for their catalytic performance in the preparation of polysubstituted imidazole derivatives **41**, **42**. The catalytic system was applied to a multicomponent reaction leading to the formation of the target compounds. The optimal conditions were identified as solvent-free, although relatively elevated temperatures in the range of 100–110 °C were required to achieve satisfactory yields of up to 96% (Scheme 38). Furthermore, the authors proposed a plausible reaction mechanism based on the experimental observations reported in the study. From a critical standpoint, while the catalyst demonstrates high efficiency in terms of product yield, the requirement for elevated temperatures may partially limit the sustainability advantages typically associated with solvent-free methodologies. In addition, although the incorporation of nitrogen-doped quantum dots is suggested to enhance catalytic activity, the study would benefit from a clearer comparison with unmodified Fe_3_O_4_ nanoparticles to explicitly demonstrate the contribution of the quantum dots to the catalytic performance. Moreover, further investigation into catalyst recyclability, structural stability, and possible leaching of active species would strengthen the evaluation of the catalyst’s practical applicability. Finally, the proposed reaction mechanism remains hypothetical and would require additional experimental or spectroscopic evidence to confirm the involvement of the suggested intermediates [104].

**Scheme 39:**
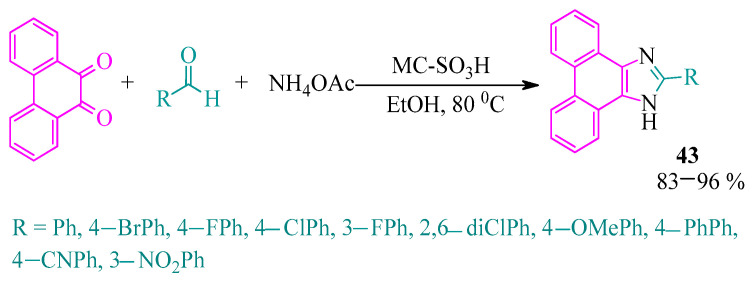
Synthesis of imidazole derivatives **43** using 9,10-phenanthrenequinone, different aldehydes and ammonium acetate under optimized reaction conditions.

The authors reported a method for the synthesis of 2,4,5-trisubstituted imidazole compounds **43** using Fe_3_O_4_/PVAm-SO_3_H nanocatalyst, which modified by coating Fe_3_O_4_ with cellulose fibers and then treating them with chlorosulfonic acid, as a catalysic system. The approach provides efficient access to the synthesis of imidazole derivatives **43** with excellent yields (83–96%) using ethanol as the solvent in a relatively short period of time (Scheme 39). Nevertheless, the practical significance of this approach remains unclear. Although catalyst recyclability was demonstrated, the limited number of cycles does not fully establish long-term stability. Thus, further studies are required to establish its general applicability [105].

**Scheme 40:**
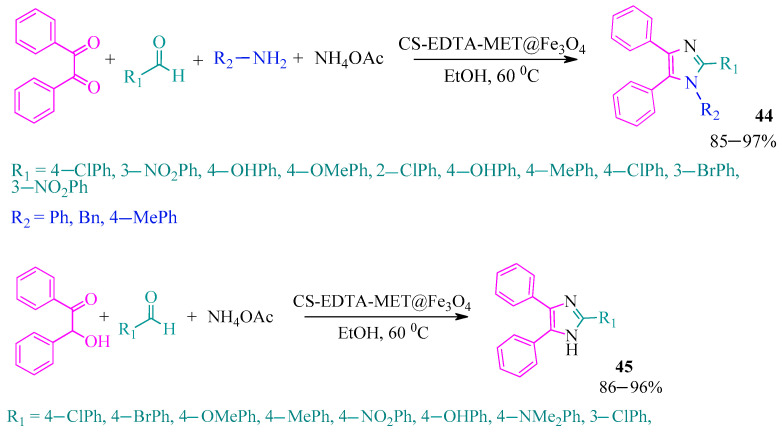
Synthesis of 2,3,4-trisubstituted and 1,2,3,4-tetrasubstituted imidazole derivatives **44**, **45** catalyzed by the CS-EDTA-MET@Fe_3_O_4_ nanocatalyst.

An innovative magnetic nanocomposite material based on methionine, ethylenediaminetetraacetic acid (EDTA), chitosan, and iron oxide (Fe_3_O_4_), designated CS-EDTA-MET@ Fe_3_O_4_, was synthesized for the synthesis of 2,4,5- and 1,2,4,5-substituted imidazole derivatives **44**, **45** (Scheme 40). The reactions were conducted in ethanol at 60 °C, yielding 11 products with yields ranging from 85% to 97%. The catalyst could be reused up to six times without loss of activity. The study also described the catalytic mechanism of the reactions and provides a significant improvement compared to previously reported methodologies [106].

The ease of catalyst recovery through filtration or magnetic separation significantly enhances the sustainability of these methodologies. Magnetic nanocatalysts, for instance, can be efficiently separated from the reaction mixture using an external magnetic field, eliminating the need for laborious filtration or centrifugation. Similarly, heterogeneous catalysts supported on polymers, silica, or other solid matrices can be recovered through simple filtration and reused in multiple reaction cycles without significant loss of activity. This recyclability not only reduces material costs but also minimizes environmental impact by decreasing the generation of catalyst-containing waste. In addition, facile recovery simplifies downstream purification of the target products, which is particularly valuable in pharmaceutical and fine chemical synthesis where residual metal contamination must be minimized. Overall, these features contribute to the development of greener, more economically viable, and scalable synthetic protocols (see Table 2) [107,108].

### 2.6. Ionic Liquids

Ionic liquids (ILs) serve as both solvents and catalysts in multicomponent imidazole synthesis, often enabling reactions under mild and solvent-free conditions. Their unique physicochemical properties, including negligible vapor pressure, high thermal stability, and tunable polarity, allow them to efficiently dissolve a wide range of substrates and facilitate key reaction steps such as carbonyl activation and condensation. In many cases, ILs promote higher reaction rates and improved product selectivity compared to conventional organic solvents. Furthermore, their dual role as solvent and catalyst can simplify reaction protocols, reducing the need for additional reagents and minimizing waste generation. Many ILs are also recyclable, allowing easy separation from products and reuse in multiple reaction cycles, which significantly enhances the sustainability of synthetic methodologies. The combination of mild conditions, operational simplicity, and environmental benefits has made ILs an increasingly attractive option for green and sustainable multicomponent reactions in heterocyclic chemistry [113,114,115].

**Scheme 41:**
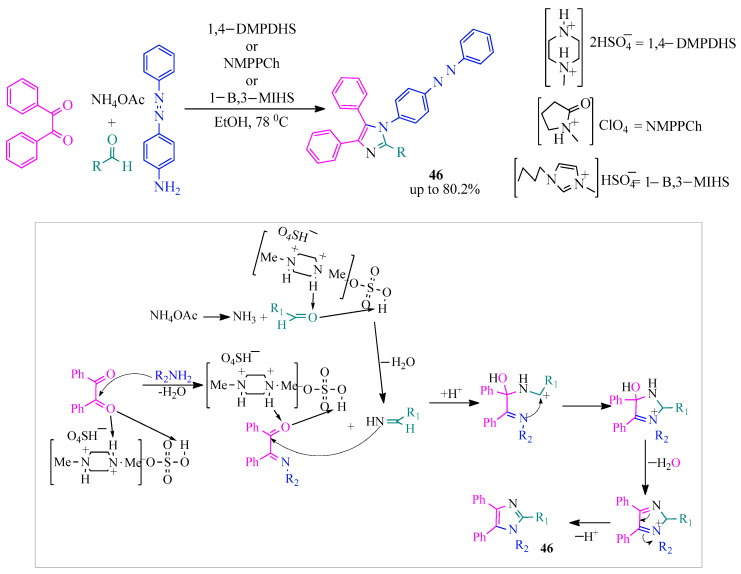
A four-component reaction scheme for tetrasubstituted imidazole **46** synthesis featuring three ionic liquids.

The performance of three ionic liquid catalysts, *N*-methylpyrrolidone perchlorate (NMPPCh), 1-butyl-3-methylimidazolium hydrogen sulfate (1-B,3-MIHS) and 1,4-dimethylpiperazine dihydrogen sulfate (1,4-DMPDHS), was investigated for the synthesis of polysubstituted imidazoles **46**. The optimal reaction conditions were determined to be 10% molar fraction of catalyst and a reaction temperature of 78 °C. Under these conditions, the imidazole derivatives **46** were isolated in good yields within a short period of time. Additionally, a theoretical analysis of the catalytic mechanism of the 1,4-dimethylpiperazine dihydrogen sulfate catalyst was conducted. This catalyst was found to activate the carbonyl groups of aldehydes and diketones, forming an imine intermediate in the reaction, as shown in Scheme 41 [116].

**Scheme 42:**
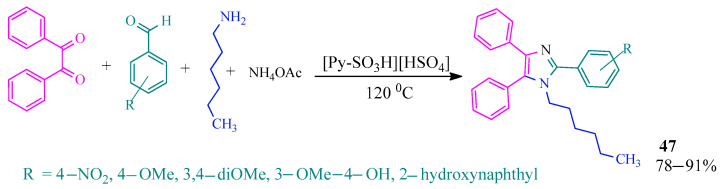
A general scheme of trisubstituted imidazole **47** synthesis using [Py-SO_3_H][HSO_4_] ionic liquid as a catalyst.

The anti-corrosive properties of several imidazole derivatives were investigated, leading to the synthesis of five triphenylhexyl imidazole compounds **47** using the ionic liquid pyridine hydrogen sulfate ([Py-SO_3_H][HSO_4_]) as a catalytic medium. The protocol enables the formation of pure products in good yields (78–91%) within a comparatively short reaction time (Scheme 42). However, the practical significance of this catalytic system remains somewhat uncertain, as ionic liquids such as [Py-SO_3_H][HSO_4_] are already widely used in acid-catalyzed transformations, which may limit the methodological novelty of the approach. Furthermore, the relatively small number of synthesized derivatives restricts a comprehensive evaluation of the structure–activity relationship related to their anti-corrosive performance. Consequently, although the reported method offers a straightforward route to triphenylhexyl imidazole derivatives, further studies would be necessary to confirm both their catalytic relevance and their effectiveness as corrosion inhibitors [117].

**Scheme 43:**
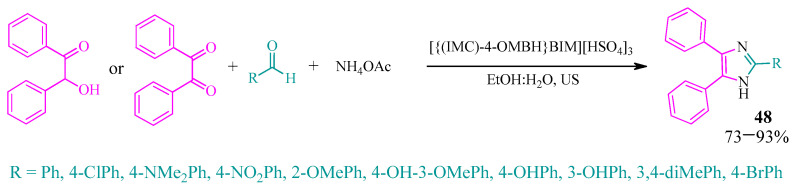
The general imidazole synthesis scheme catalyzed by the [{(IMC)-4-OMBH}BIM][HSO_4_]_3_ ionic liquid.

The authors reported a method for the synthesis of 10 2,4,5-trisubstituted imidazole compounds **48** in excellent yields of up to 99% under ultrasonic irradiation (as shown in Scheme 43) using a novel ionic liquids, 1,1′-{3,3′-(3-(1*H*-imidazol-3-ylcarbonyl)-4-oxo-cyclohexa-2,5-diene-1-ylidene)-bis(6-hydroxy-benzoyl)bis-(1*H*-imidazole-3-ium)}-trihydrogen sulfate (IMC-4-OMBH[HSO_4_]_3_) as a catalyst. Despite the high yields achieved, it is unclear whether the catalyst offers a significant improvement over previously reported methods. Therefore, further research is required to determine its general applicability [118].

**Scheme 44:**
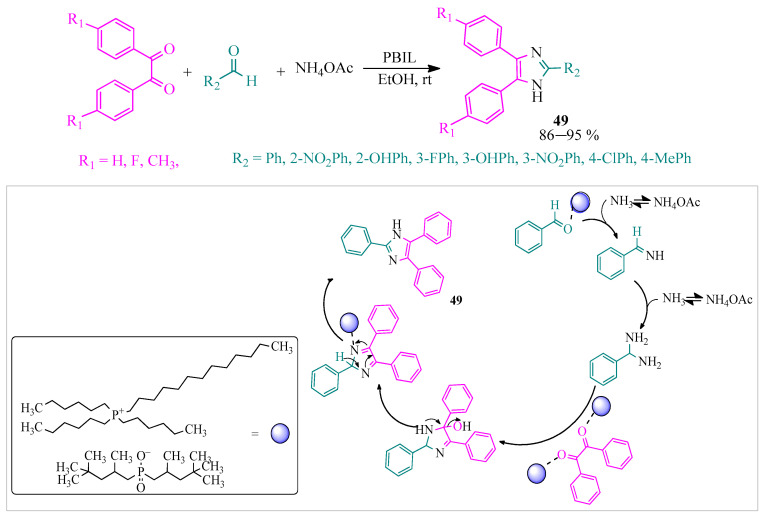
Trisubstituted imidazole **49** synthesis schematic with the help of PBIL ionic liquid.

A protocol based on a catalytic amount of the ionic liquid PBIL (trihexyltetradecylphosphonium bis(2,4,4-trimethyl-pentyl)phosphinate) was proposed for the preparation of the synthesis of 2,4,5-triarylimidazoles **49**. The method affords the desired products in high yields (>85%) by dissolving the reaction mixture in ethanol at room temperature (Scheme 44). Moreover, more extensive recycling experiments would be required to confirm catalyst durability [119].

**Scheme 45:**
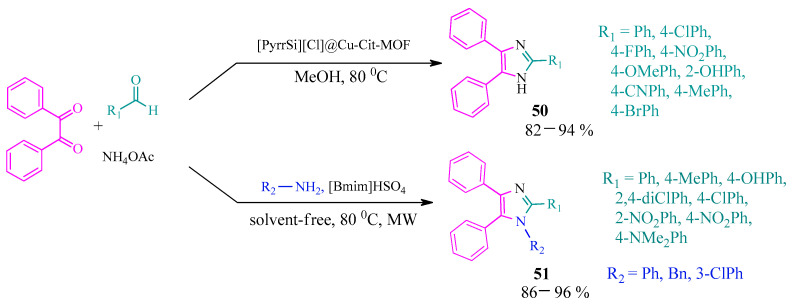
A scheme of using [PyrrSi][Cl]@Cu-Cit-MOF and [Bmim]HSO_4_ ionic liquids as catalysts for imidazole **50**, **51** synthesis.

**Scheme 46:**
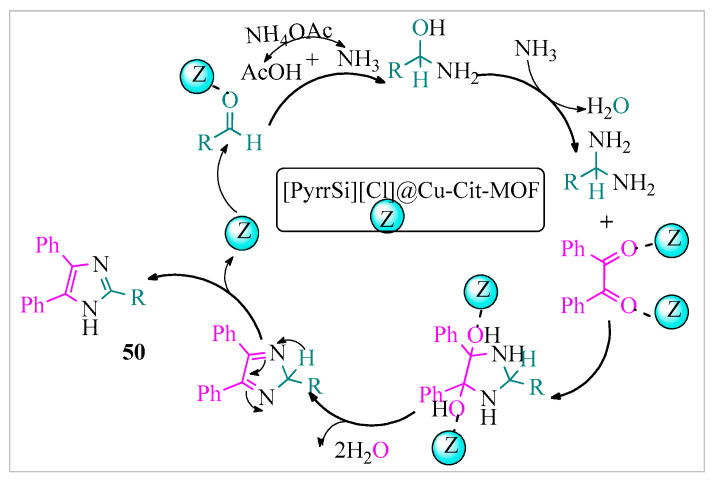
Plausible reaction mechanism of using [PyrrSi][Cl]@Cu-Cit-MOF for synthesis of imidazoles **50**.

A simple method for the preparation of a new silica-based pyrrhylene ionic liquid supported on a copper–citrate organometallic framework (PyrrSiCl@Cu-Cit-MOF) was described. This ionic liquid has been tested as a catalyst for a multicomponent reaction, leading to the formation of trisubstituted imidazoles **50** (Scheme 45 and Scheme 46). Following the instructions provided in the research article (addition of methanol and heating to 80 °C), various imidazole derivatives **50** were obtained in excellent yields (82–94%) within a short period of time. In addition, under the specified conditions, this ionic liquid allows the use of a variety of aldehydes as starting materials, and the catalyst can be easily recovered from the reaction mixture through simple filtration and reused up to six times with no significant loss of catalytic activity [120].

**Scheme 47:**
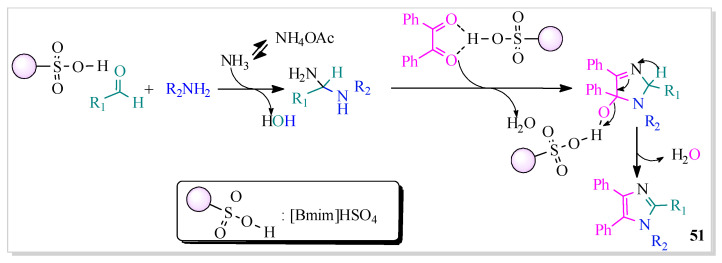
The proposed reaction mechanism of the imidazole **51** synthesis using [Bmim]HSO_4_.

A study has reported the synthesis of 11 new tetrasubstituted imidazole derivatives **51** using the ionic liquid 1-butyl-3-methylimidazolium hydrogen sulfate ([Bmim]HSO_4_) as a catalyst. The reactions were performed under solvent-free conditions in a microwave reactor at a power of 100 W and a temperature of 80 °C, affording excellent yields of up to 96% (Scheme 47). The authors proposed that the catalytic role of [Bmim]HSO_4_ involves the activation of the aldehyde and 1,2-diketone substrates, which subsequently react with an amine and ammonium acetate to form a diamine intermediate, followed by cyclization to generate the five-membered imidazole ring. From a critical perspective, although the methodology provides high yields under relatively mild microwave conditions, the requirement for specialized microwave equipment may limit its broader practical applicability compared with conventional thermal methods. Furthermore, while ionic liquids such as [Bmim]HSO_4_ are often described as “green” catalysts, their environmental advantages remain debatable due to potential issues related to synthesis, toxicity, and recyclability. The study would also benefit from a more detailed investigation of catalyst recovery and reuse, as well as comparative experiments with other Brønsted acid catalysts to clearly demonstrate the specific advantages of [Bmim]HSO_4_. Finally, the proposed mechanism remains tentative and would require additional experimental evidence to confirm the activation mode of the substrates and the formation of the suggested intermediates [121].

Their negligible vapor pressure and recyclability make these systems attractive alternatives to conventional organic solvents. In addition, their high thermal and chemical stability allows reactions to be conducted over a wide range of temperatures without significant solvent degradation. Many ionic liquids can also be readily recovered from reaction mixtures by simple extraction or phase separation and reused in multiple reaction cycles with minimal loss of catalytic activity. These features not only reduce solvent consumption and waste generation but also contribute to improved process sustainability and economic efficiency. Consequently, ionic liquids have gained considerable attention as environmentally benign reaction media for a variety of organic transformations, including multicomponent synthesis of heterocyclic compounds such as imidazoles [122,123].

**Figure 2:**
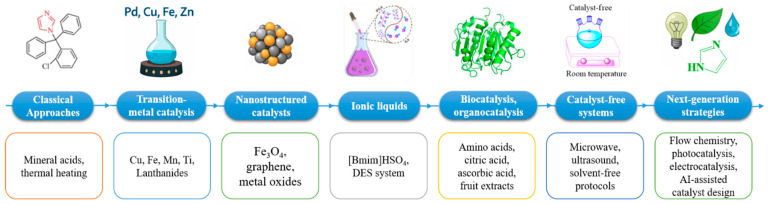
Evolution of catalytic strategies for imidazole synthesis.

The development of synthetic methods for the production of imidazole derivatives has undergone significant changes over the past few decades. In the early stages, methods relied primarily on thermal activation and acid catalysis using mineral acids, which often required harsh reaction conditions and generated large amounts of waste. Subsequent developments have seen the introduction of transition metal catalysts and nanostructured catalysts, which have improved reaction efficiency and the range of substrates that can be used. More recently, there has been an increased focus on sustainable synthetic techniques, such as those using ionic liquids, deep eutectic solvents, organocatalysts, biologically derived catalysts, and catalyst-free methods. This overall shift in catalytic platforms reflects a move away from purely efficiency-driven approaches towards more environmentally friendly and resource-conserving methods (see Figure 2).

As illustrated in Figure 2, current research is increasingly focusing on balancing catalytic activity with sustainability concerns. While transition metal and nanostructured catalysts often exhibit superior activity and broader substrate tolerance, organocatalytic, biobased, and catalyst-free systems provide important advantages in terms of reduced toxicity and environmental friendliness. Consequently, future developments in imidazole synthesis are expected to prioritize catalytic systems that combine high efficiency, recyclability, scalability, and adherence to green chemistry principles simultaneously.

To provide a clearer overview of the diverse catalytic strategies employed in imida-zole synthesis, the main methodological approaches are summarized in Table 3. These include catalyst-free systems, organocatalysis, transition metal catalysis, nanostructured catalysts, and ionic-liquid-based methodologies. Each strategy offers distinct advantages in terms of efficiency, substrate scope, and sustainability. For instance, catalyst-free approaches eliminate issues associated with catalyst contamination, whereas metal-catalyzed systems often provide higher reaction rates and broader functional group tolerance. Meanwhile, nanocatalysts and ionic liquids offer improved recyclability and operational simplicity, making them attractive for green synthetic applications. The comparison highlights how the selection of an appropriate catalytic system depends strongly on the desired substitution pattern, reaction conditions, and practical considerations.

## 3. Other Methods for Synthesizing Imidazole Derivatives

In addition to the Debus–Radziszewski reaction described above, several alternative approaches have been developed for the synthesis of structurally diverse imidazole derivatives. The following sections focus on methods based on two- and three-component reactions, which have attracted considerable attention due to their operational simplicity and efficiency.

### 3.1. Two-Component Reactions

**Scheme 48:**
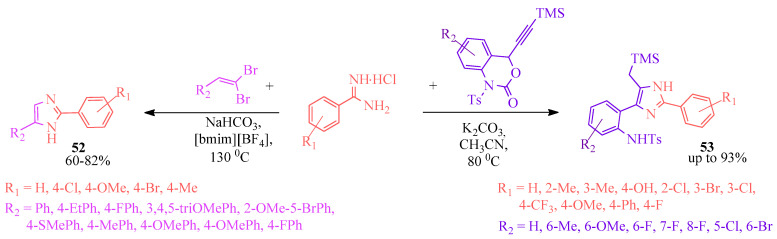
Two-component synthesis of imidazoles using amidines as one of the reagents.

**Scheme 49:**
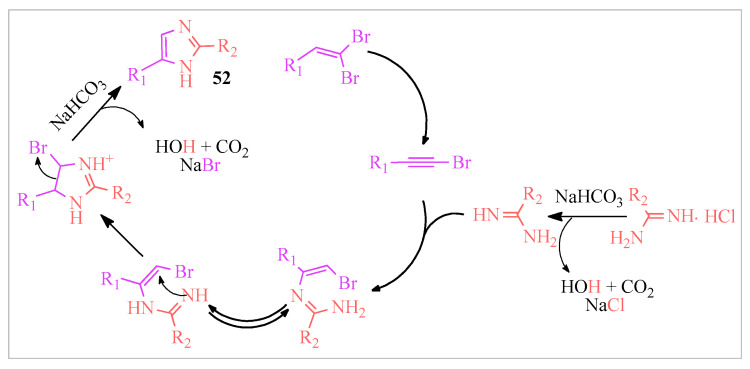
Mechanism of the reaction between gem-dibromovinylarenes and amidines, yielding **52**.

The authors investigated a method for the synthesis of 2,5-disubstituted imidazole compounds **52** based on the reaction of gem-dibromovinylarenes with amidines in the presence of a NaHCO_3_/1-butyl-3-methylimidazolium tetrafluoroborate ([bmim][BF_4_]) ionic liquid system under aerobic conditions at 130 °C (Scheme 48 and Scheme 49). Using this protocol, 26 compounds **52** were synthesized, with the maximum isolated yield reaching 82%. The protocol produces the desired products under mild conditions, but several limitations should be taken into account when evaluating the results. Specifically, no comparison was made with previously reported catalytic systems. Although the method demonstrates good yields and a broad substrate scope, the requirement for relatively high reaction temperatures (130 °C) may limit its energy efficiency and scalability. Overall, while the catalyst demonstrates promising activity, additional research is required to determine its general applicability and the mechanisms involved [134].

**Scheme 50:**
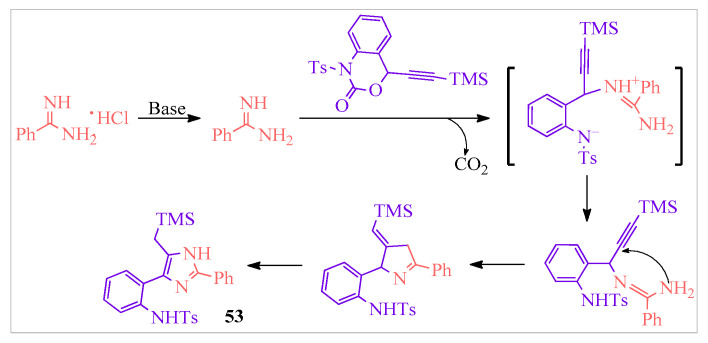
Mechanism of imidazole **53** synthesis technique through the decarboxylating [3+2]-annulation reaction.

A method for the preparation of trisubstituted imidazole derivatives **53**, by the decarboxylating [3+2]-annulation reaction between trimethylsilylethynylbenzoxazinanones and benzimidamide hydrochloride in the presence of a base under relatively mild conditions was reported (Scheme 48 and Scheme 50). The optimum reaction conditions were determined experimentally and include the use of two equivalents of K_2_CO_3_ as the base, CH_3_CN as a solvent, and heating at 80 °C for five hours. The authors synthesized 10 products **53** by varying ethynylbenzoxazinanones, with a maximum yield of 86% and, additionally, 17 products **53** by modifying the benzimidamide hydrochlorides with a maximum yield of 93%. Finally, the proposed reaction mechanism, although chemically plausible, remains speculative and would benefit from further experimental evidence, such as kinetic studies or the identification of key intermediate species [135].

**Scheme 51:**
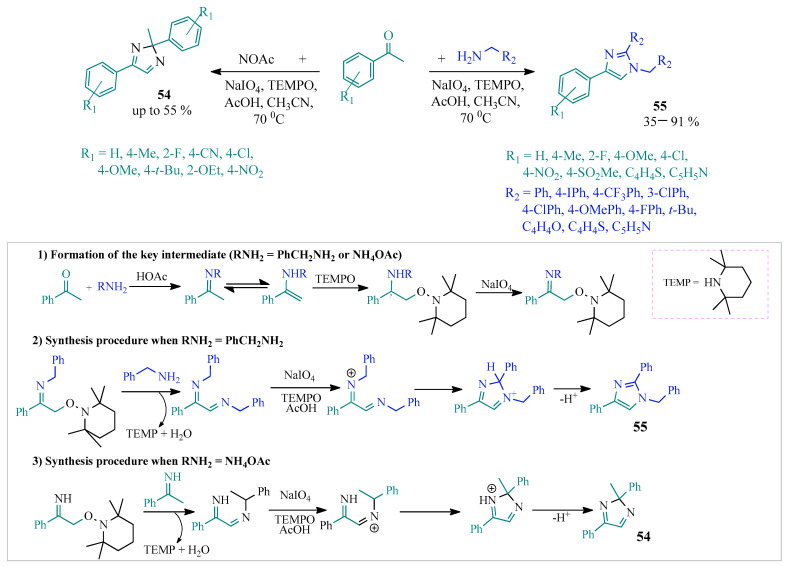
Synthesis of imidazole derivatives **54**, **55** via a two-component reaction with the proposed mechanism.

A protocol based on oxidative cyclization of arylmethyl ketones with amines in the presence of (2,2,6,6-tetramethylpiperidinyl-*N*-oxyl) derivative (TEMPO) and NaIO_4_ was proposed for the preparation of polysubstituted 1*H*-imidazoles **55**. The method affords the desired products in good yields reaching 91%. This strategy offers to synthesize products containing 2*H*-imidazoles **54** by replacing amines with ammonium acetate. The applicability of this method was verified through the large-scale synthesis and Sonogashira coupling of imidazole functionalization. Mechanistically, the α-TEMPO-enamine adduct appears to be a key reaction intermediate (Scheme 51) [136].

**Scheme 52:**
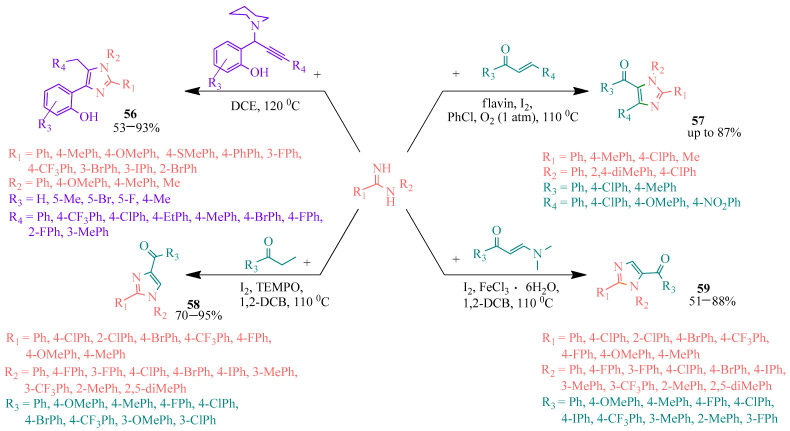
Four imidazoles synthesis protocols sharing amidines as one of the reagents.

**Scheme 53:**
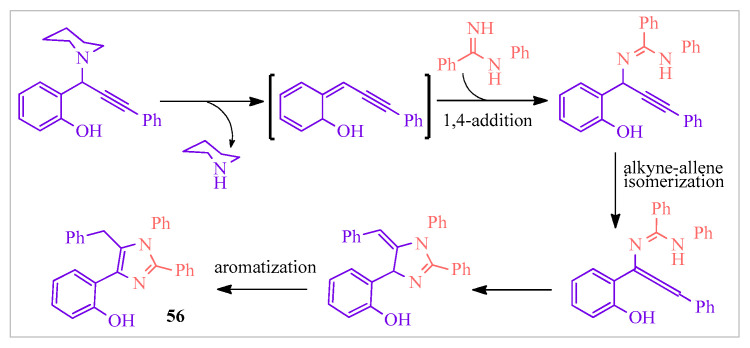
Mechanism of the cascade 1,4-conjugate addition protocol, yielding **56**.

The reported strategy based on 1,4-conjugate addition for the synthesis of 1,2,4,5-tetrasubstituted imidazoles **56** involves the use of propagylamines and amidines as reagents and dichloroethane (DCE) as a solvent (Scheme 52 and Scheme 53). Using the proposed method, the authors succeeded in synthesizing a series of imidazole compounds **56**, with a maximum yield of 87%. Furthermore, the gram-scale synthesis and late-stage transformations make this protocol suitable for the preparation of a variety of *N*-heterocycles [137].

**Scheme 54:**
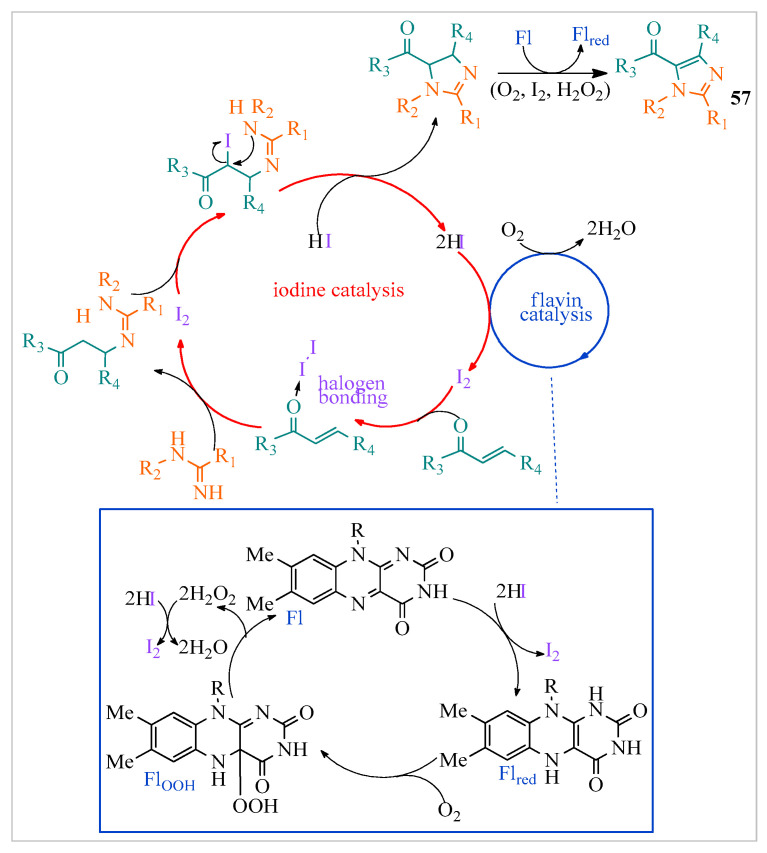
Mechanism of polysubstituted imidazole derivative **57** synthesis protocol catalyzed using flavin–iodine system.

In one of the reported studies on new approaches to the synthesis of imidazole derivatives, an economical method for the preparation of tetrasubstituted imidazole compounds **57** via the aerobic cross-dehydrogenative coupling of chalcones and amidines was described (Scheme 52). For this transformation, the authors employed a catalytic system based on iodine and flavin. The reaction was carried out in chlorobenzene under an oxygen atmosphere (1 atm) with heating to 110 °C. Under these conditions, the desired tetrasubstituted imidazoles were obtained in high yields (up to 87%). In addition, the authors proposed a possible mechanism explaining the catalytic role of the iodine–flavin system, which is illustrated in Scheme 54. Although the methodology is presented as economical and makes use of molecular oxygen as a terminal oxidant, several limitations should be noted. The requirement for relatively high reaction temperatures and the use of chlorobenzene, a solvent associated with environmental and safety concerns, may reduce the overall sustainability of the process. Furthermore, the catalytic system involving iodine and flavin derivatives may raise questions regarding catalyst stability and recyclability, aspects that require further investigation to assess the practical applicability of the method. Therefore, while the proposed reaction mechanism provides a reasonable explanation for the observed transformation, it remains hypothetical and would benefit from additional experimental evidence, such as mechanistic studies [138].

**Scheme 55:**
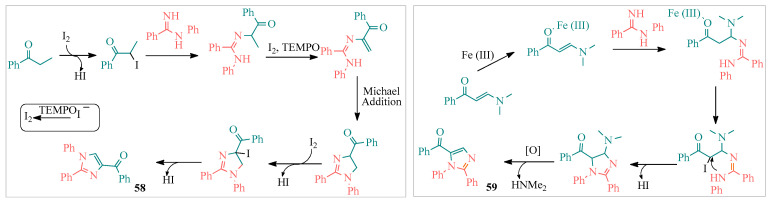
Mechanism of polysubstituted imidazole derivative **58**, **59** synthesis protocols via iodine-catalyzed [3+2] cycloaddition reactions.

Two techniques for the selective synthesis of 1,2,4- and 1,2,5-trisubstituted imidazoles **58**, **59** via iodine-catalyzed [3+2] cycloaddition reactions were reported. The first technique utilizes amidines and propiophenones as substrates, iodine in a catalytic amount (0.5 equivalent), and TEMPO (1.5 equivalent) as an additive (Scheme 52). The reaction takes place in an oil bath at 100 °C using 1,2-dichlorobenzene (1,2-DCB) as a solvent. To test the feasibility of this technique, 39 products **58** were synthesized, the lowest yield of which was 70%. The second technique is a slightly modified version of the first technique mentioned above (Scheme 51). Amidines and enaminones serve as starting reagents, and the catalyst is the system I_2_ (0.5 equivalent), FeCl_3_∙6H_2_O (0.4 equivalent). The solvent remained the same, but the temperature was increased to 110 °C. Forty-two different pro-ducts **59** were obtained using this technique in yields ranging from 51% to 88%. The authors of the cited study also conducted an analysis of the most probable reaction mechanisms for the synthesis of imidazole compounds (Scheme 55) [139].

**Scheme 56:**
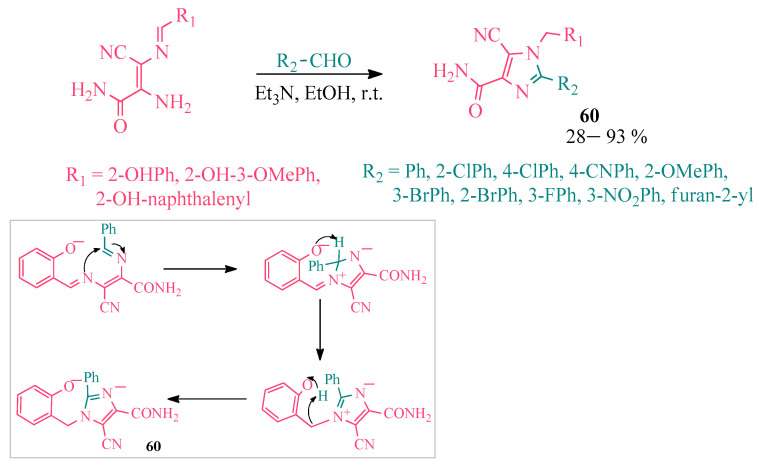
Acquisition of tetrasubstituted imidazoles **60**.

A recent study described a method for the synthesis of imidazole derivatives **60** from aryl aldehydes and benzylidene imines derived from diaminomaleonitrile (Scheme 56). The reaction proceeds at room temperature in ethanol using triethylamine as a base. Using this protocol, a total of 18 products **60** were synthesized, with yields ranging from 28% to 93%. The method offers several advantages, including mild reaction conditions and the use of a relatively simple catalytic system. However, the wide variation in product yields suggests that the reaction efficiency may be strongly dependent on the electronic or steric properties of the substrates. In addition, the good yields obtained for some derivatives indicate that further optimization of the reaction conditions may be required to improve its general applicability. Moreover, although the procedure operates under mild conditions, the scope of the reaction could be further expanded by testing a broader range of functionalized aldehydes and imines [140].

**Scheme 57:**
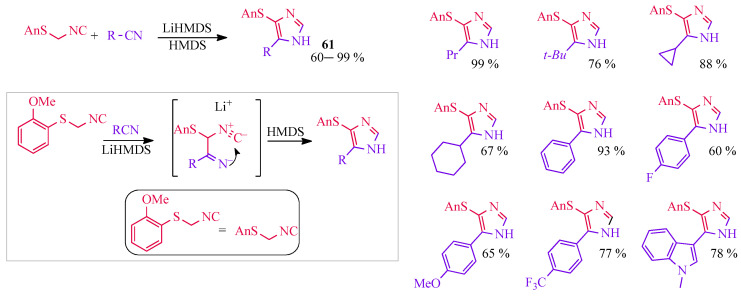
Synthesis of substituted imidazole compounds **61**.

Certain types of substituted imidazoles **61** can be readily prepared by the condensation of anisylsulfanylmethyl isocyanide with nitrogen-containing π-electrophiles, such as nitriles. A paper devoted to such transformations describes a procedure for the synthesis of 4,5-disubstituted imidazole compounds using the above-mentioned reagents in the presence of lithium hexamethyldisilazide (LiHMDS) base and its conjugate acid, hexamethyldisilazane (Scheme 57). During their experiments, the authors of the paper succeeded in synthesizing twelve products **61** with an average yield of 75% [141].

**Scheme 58:**
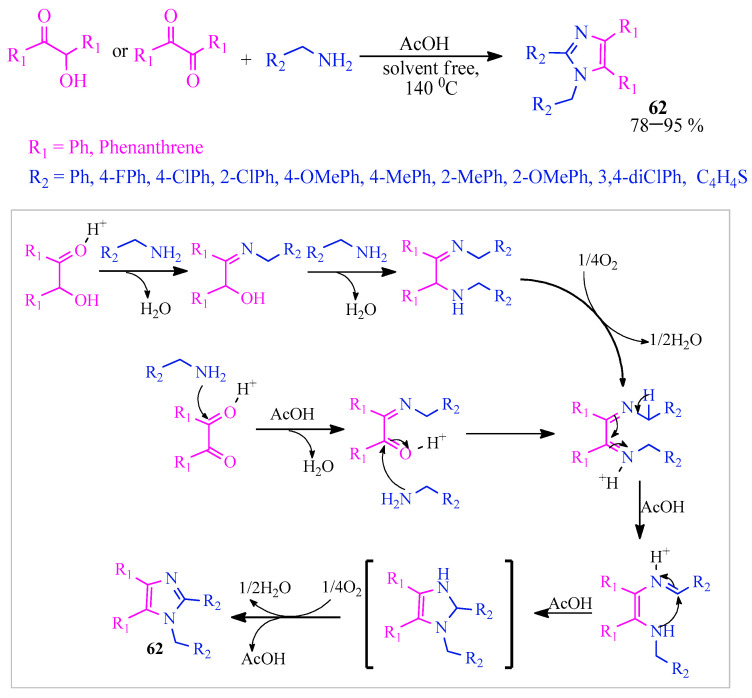
Tetrasubstituted imidazole **62** synthesis with the reaction mechanism.

A relatively simple procedure for the synthesis of 1,2,4,5-tetrasubstituted imidazole derivatives **62** via the solvent-free reaction of 1,2-diketones with amines (Scheme 58) was proposed in a recently published paper. The solvent-free nature of the reaction represents a notable advantage from the standpoint of green chemistry and operational simplicity. However, the study provides limited information regarding the scope and limitations of the method, particularly with regard to the tolerance for different functional groups in starting materials. Additionally, although high yield was reported, further data on scalability and reproducibility of the reaction would be beneficial for assessing the practical feasibility of the procedure [142].

### 3.2. Three-Component Reactions

**Scheme 59:**
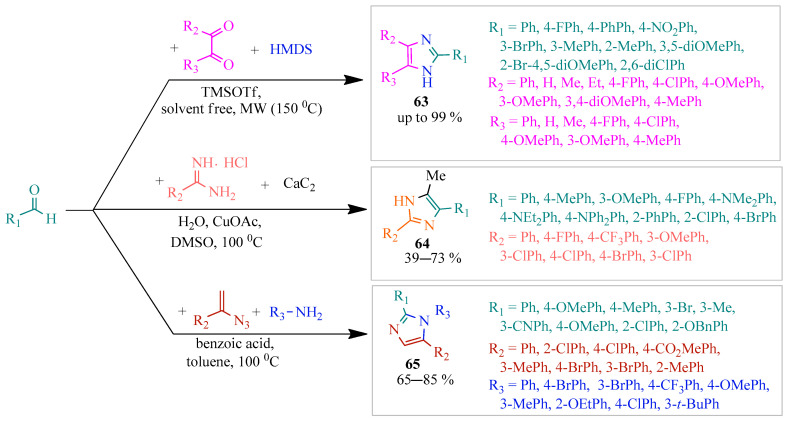
Polysubstituted imidazole **63**–**65** syntheses via three-component reactions that share aldehydes as the common reagent.

**Scheme 60:**
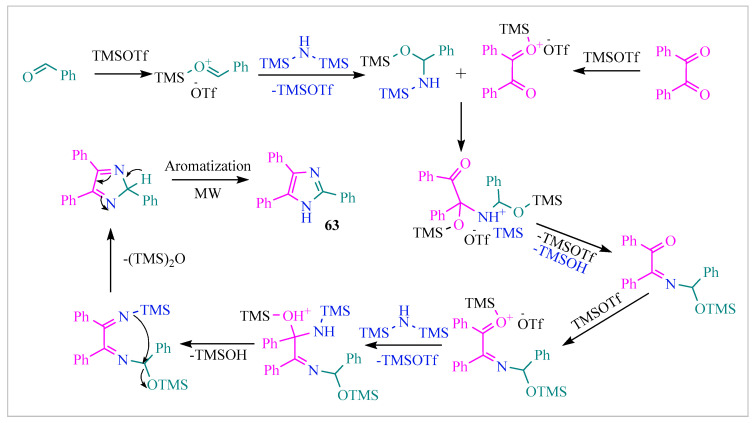
Three-component trisubstituted imidazole **63** synthesis reaction mechanism.

A Lewis acid-catalyzed protocol employing catalytic amounts of trimethylsilyl trifluoromethanesulfonate (TMSOTf) has been proposed for the synthesis of 2,4,5-trisubstituted imidazoles **63** from 1,2-diketones, aldehydes, and bis(trimethylsilyl)amine (HMDS) (Scheme 59 and Scheme 60). The method affords the desired products in high yields under solvent-free microwave conditions at elevated temperature (150 °C), with a broad substrate variation leading to up to 29 derivatives from aldehyde variation and 13 from diketone modification. While the reported yields are notably high (up to 99%), the requirement for harsh microwave heating conditions and elevated temperature may limit the practical scalability and energy efficiency of the protocol. Furthermore, although a detailed mechanistic rationale for Lewis acid activation was proposed, the study appears to rely primarily on postulated reaction pathways without direct experimental validation, such as kinetic analysis or isolation of intermediates. Overall, while the method demonstrates efficient access to substituted imidazoles, its operational intensity and mechanistic assumptions suggest that further comparative and mechanistic studies are required to fully establish its synthetic robustness [143].

**Scheme 61:**
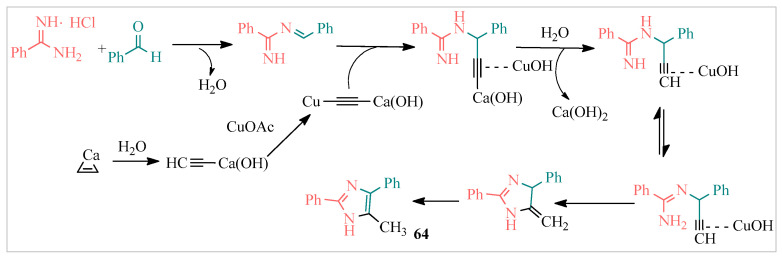
Mechanism of a three-component imidazole **64** synthesis via A3-coupling and cyclization reactions.

Recently, a new and efficient strategy was developed for the synthesis of 5-methyl-2,4-diaryl-1*H*-imidazoles **64**. The protocol affords the desired products based on copper(I) salt-catalyzed A3-coupling and cyclization reactions using benzamidine, benzaldehyde, and calcium carbide (Scheme 59 and Scheme 61). The successful synthesis of 34 products **64** in a maximum yield of 73% was reported. However, the absence of comparative studies with established catalysts makes it difficult to assess the true efficiency of the system [144].

**Scheme 62:**
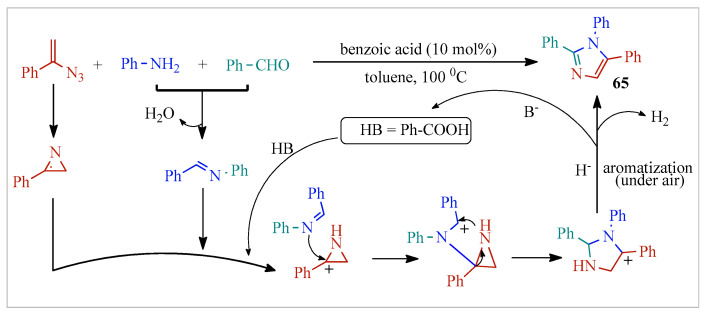
Mechanism of 1,2,5-trisubstituted imidazole derivatives **65** from vinyl azides with aromatic aldehydes and amines.

An efficient technique for preparing 1,2,5-trisubstituted imidazole derivatives **65** was proposed via the reaction of vinyl azides with aromatic aldehydes and amines (Scheme 59 and Scheme 62). This reaction occurs in the presence of Brønsted acid as a catalyst and solvent at 100 °C. In the paper describing this technique, the authors used benzoic acid and toluene and determined the optimal reagent ratio to maximize the yield. This ratio is 1.5 equivalents of vinyl azide, 1.0 equivalent of aromatic aldehyde, and 1.0 equivalent of aromatic amine. Under these conditions, 36 different products **65** were obtained in good yields (65–85%). While the reported yields are notably high (up to 85%), the requirement for the heating temperature may limit the practical scalability and energy efficiency of the protocol [145].

**Scheme 63:**
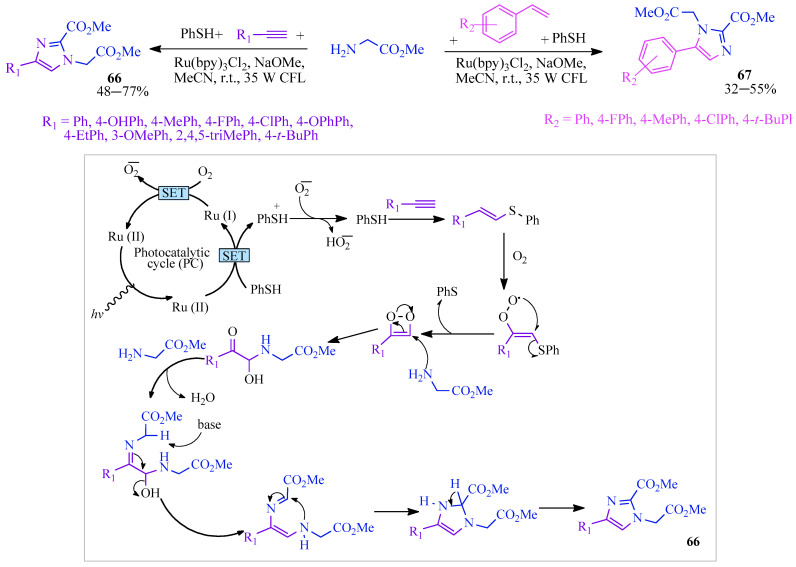
Synthesis of trisubstituted imidazoles **66**, **67** through photoreduction of terminal alkynes and styrenes with mechanism present.

The synthesis of trisubstituted imidazoles **66**, **67** through the photoreduction of terminal alkynes and glycine methyl ester using the required catalysts was analyzed and presented as a simple procedure (Scheme 63). Along with 22 products **66** obtained in 48–77% yields using terminal alkynes, five products **67** were also obtained from a similar reaction (with a maximum yield of 55%) using substituted styrenes instead of alkynes. In the cited article, the authors propose using a 35 W compact fluorescent lamp and a mixture of tris(bipyridine)ruthenium(II) chloride (Ru(bpy_3_)Cl_2_) and thiophenol in a 1:10 molar ratio as a photocatalyst for photoreduction. Although the methodology demonstrates the potential of visible light photoredox catalysis for imidazole synthesis, several limitations can be identified. Furthermore, the relatively average yields observed for styrene substrates suggest a limited substrate scope and lower efficiency compared with terminal alkynes. Overall, while the current strategy is innovative and easy to implement, further optimization and investigation of metal-free options would enhance its practicality [146].

**Scheme 64:**
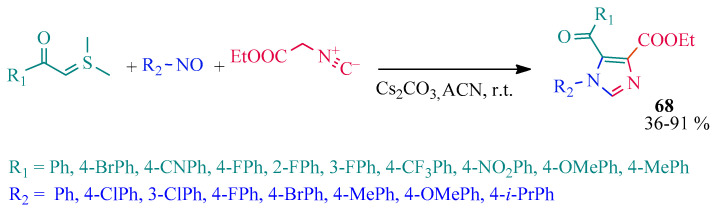
Synthesis of polysubstituted 2–imidazolones **68**.

A three-component reaction involving sulfur ylide, nitrobenzene, and isonitrile compounds was proposed for the synthesis of polysubstituted 2-imidazolones **68**. The reaction proceeds under mild conditions without the use of a catalyst, using anhydrous acetonitrile as a solvent and Cs_2_CO_3_ as a base (Scheme 64). Having obtained 22 products **68** in good yields with the maximum yield of 91%, the following reagent ratio was established to increase the yield: 1.5 equivalents of sulfur ylide, 1.5 equivalents of nitrosobenzene, and 1.0 equivalent of isonitrile ethyl acetate. Despite these advantages, the practical implications of this approach remain uncertain. Without comparison to known catalytic protocols, the claimed benefits remain only partially supported [147].

**Scheme 65:**
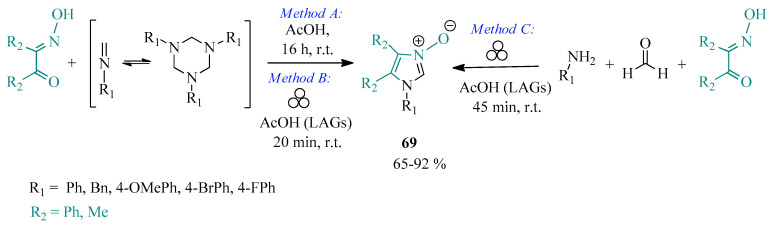
Two- and three-component syntheses of 2–unsubstituted imidazole *N*–oxide **69** in solution (Method A) and under mechanochemical activation (Methods B and C).

2-unsubstituted imidazole-N-oxides **69** were obtained using the mechanochemical method of ball grinding. Upon condensation of hydroxyiminoketones and formaldimines in acetic acid as a solvent (method A) or using a mechanochemical approach (methods B and C), where grinding was carried out in a ball mill for 20–45 min at room temperature, imidazole-N-oxides **69** were isolated with a yield of 34–92% (Scheme 65). Furthermore, these compounds can be, for example, converted to enolizable imidazole-2-thions through a sulfur transfer reaction or to non-enolizable imidazole-2-thions through in situ sulfurization of generated imidazole-2-ylidenes [148].

These alternative methodologies often allow for the rapid construction of imidazole frameworks under mild conditions and may reduce the number of purification steps required. However, despite these benefits, several limitations still exist. In some instances, multicomponent reactions may suffer from a narrow substrate scope, decreased selectivity, or the formation of byproducts arising from competing pathways. Additionally, many reported methods rely on high temperatures, specialized catalysts, or unfriendly reaction conditions, which can limit their practicality and environmental sustainability. Therefore, it remains an important goal in contemporary heterocyclic chemistry to continue developing more efficient, selective, and eco-friendly methods for synthesizing imidazole derivatives.

## 4. Practical Applications of Imidazole Derivatives

**Figure 3:**
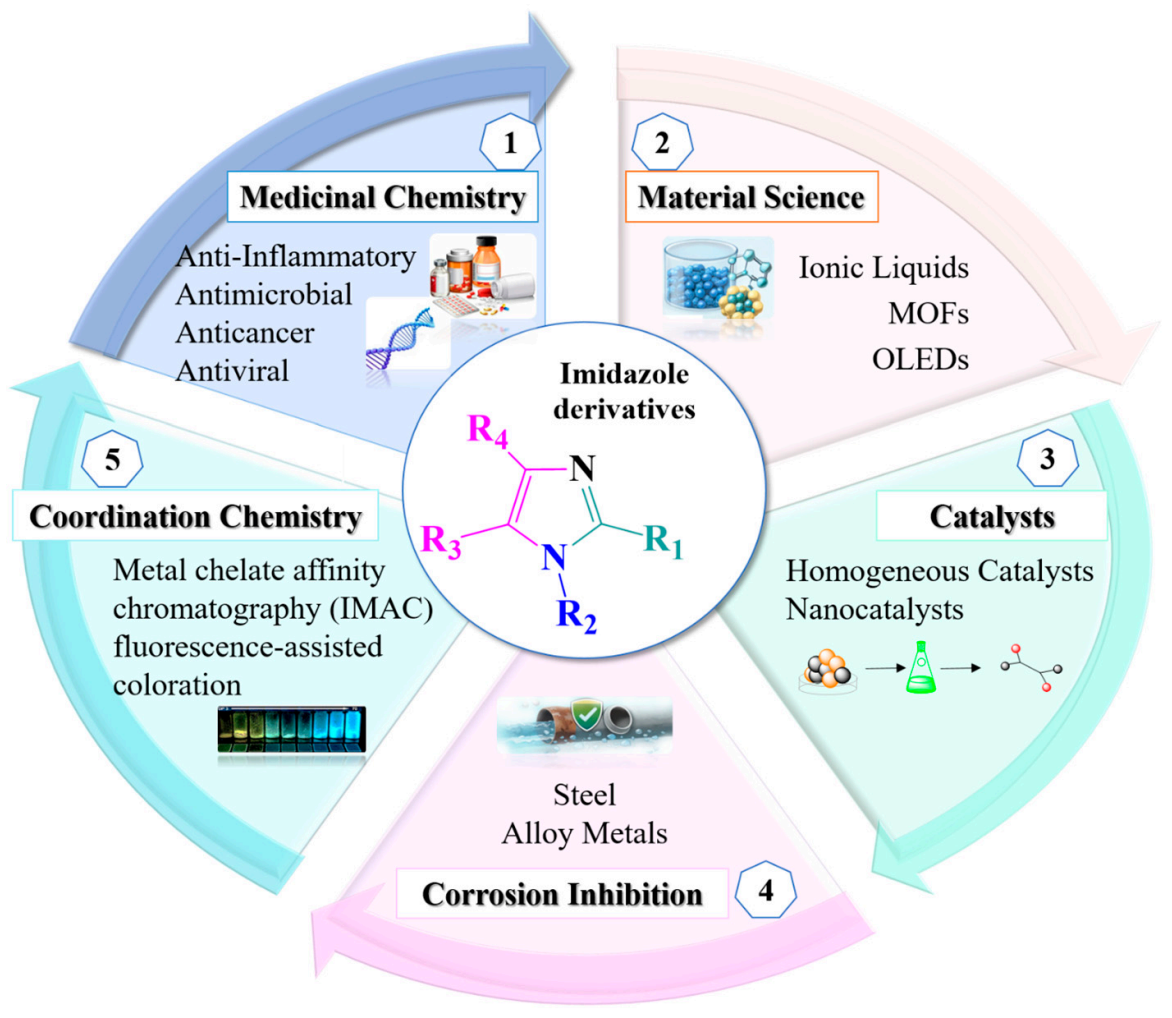
Practical applications of imidazole derivatives.

The broad applicability of imidazole derivatives stems from the unique electronic and coordination properties of the imidazole ring, which allow effective interactions with biological targets and metal surfaces. As summarized in Table 4 and Figure 3, numerous studies have demonstrated that appropriately substituted imidazole frameworks exhibit significant antimicrobial, anticancer, and anti-inflammatory activities, highlighting their importance in medicinal chemistry. Beyond pharmaceutical applications, these compounds also play an important role in materials science and industrial chemistry. In particular, imidazole derivatives have been widely employed as corrosion inhibitors for steel and other metal alloys due to their strong adsorption on metal surfaces. Furthermore, the tunable electronic properties of the imidazole scaffold have enabled its incorporation into functional materials such as ionic liquids, coordination complexes, and optoelectronic systems. This versatility underscores the importance of developing efficient synthetic methodologies capable of rapidly generating structurally diverse imidazole derivatives.

## 5. Future Directions in Imidazole Synthesis

The development of imidazole derivative synthesis continues to progress towards more sustainable, efficient, and application-oriented methods. While significant progress has been made through the use of multicomponent reactions, advanced catalytic systems, and green chemistry techniques, several challenges remain before many of these methods can be widely implemented on an industrial scale.

A key future area of focus is the development of biobased and environmentally friendly catalytic systems. Natural organic acids, amino acids, biopolymers, and biomass-derived materials offer promising alternatives to conventional catalysts due to their low toxicity, biodegradability, and renewability. Future research should aim not only to improve their catalytic performance but also to understand the underlying mechanisms that govern their activity.

Deep eutectic solvents (DESs) are expected to play a growing role in sustainable imidazole synthesis. Compared to conventional ionic liquids, they are generally less expensive and easier to prepare, while often exhibiting improved environmental profiles. However, further research is needed to systematically investigate their physicochemical properties, substrate compatibility, and long-term recyclability in order to establish general guidelines for DES-mediated imidazole synthesis [162,163].

Another promising area of research is the integration of continuous flow technologies with multicomponent imidazole synthesis. Continuous flow chemistry offers significant benefits in terms of process safety, heat and mass transfer, reaction control, and scalability. The combination of flow reactors and recyclable catalytic systems could facilitate the transition from laboratory-scale to industrial-scale production [164].

Emerging activation technologies, such as photocatalysis and electrocatalysis, present additional opportunities for developing energy-efficient synthetic methods. These approaches could potentially enable the formation of imidazole structures under milder conditions, while reducing the need for stoichiometric oxidizing agents or harsh reaction conditions. However, their application in imidazole synthesis has been relatively underexplored, and further research is warranted.

From a mechanistic standpoint, future research should place a greater emphasis on the rigorous elucidation of reaction pathways. Despite the numerous catalytic systems that have been reported in recent years, our mechanistic understanding of these systems often remains limited to mere reaction proposals. Integrating kinetic studies, in situ spectroscopic techniques, computational chemistry, and density functional theory (DFT) calculations would be essential for establishing structure–activity relationships and guiding the rational design of next-generation catalysts.

An important challenge that has not been sufficiently addressed is the lack of standardized protocols for benchmarking catalytic performance in imidazole synthesis. Comparisons between different catalytic systems are often complicated by variations in substrate selection, catalyst loading, reaction conditions (temperature, solvent), and product isolation methods. To address this challenge, future studies should incorporate standardized metrics for green chemistry, such as catalyst productivity, reusability, atom efficiency, energy consumption, and mass intensity of the process. These metrics would allow for a more objective evaluation of catalytic technologies and the identification of more sustainable synthetic methods.

Furthermore, the increasing availability of artificial intelligence (AI) and machine learning (ML) tools may significantly expedite catalyst discovery and reaction optimization processes. Data-driven approaches can assist in predicting the performance of catalysts, identifying optimal reaction conditions, and reducing the experimental workload, thus contributing to the more efficient development of sustainable synthesis methods [165].

Overall, future developments in imidazole synthesis are likely to depend on the successful implementation of green chemistry principles, a better understanding of the underlying mechanisms, scalable process technologies, and the use of digital tools for catalyst design. These advances are expected to enhance the availability of imidazole-based derivatives for use in medicinal chemistry, materials science, agricultural chemicals, and functional molecular systems.

## 6. Conclusions and Outlook

Recent advances in the synthesis of imidazole derivatives have greatly expanded the range of methods available for the creation of these important heterocyclic frameworks. The continuous development of multicomponent reactions, especially those based on the Debus–Radziszewski condensation, has allowed for the efficient preparation of a diverse range of tri- and tetrasubstituted imidazoles under increasingly environmentally friendly and straightforward conditions. As this review demonstrates, catalyst-free approaches, organocatalysis, metal-catalyzed reactions, nanostructured catalysts, and ionic liquids all offer unique advantages in terms of reaction efficiency, reactivity towards a wide range of substrates, selectivity, and sustainability. A clear trend that has emerged from recent literature is a shift away from purely yield-based methodologies towards more sustainable synthetic approaches that emphasize recyclability of catalysts, minimization of waste, reduction in energy consumption, and use of environmentally friendly reaction media. In this context, organocatalysts, biologically derived catalytic systems, and ionic liquids have garnered significant attention as alternatives to traditional catalytic platforms. Additionally, nanostructured catalysts have shown remarkable catalytic activity and potential for recovery, particularly when combined with magnetic separation techniques. However, it is challenging to accurately assess the practical benefits of many new catalytic systems due to a lack of comparative analysis with established methods. Despite significant progress, there are several significant challenges that continue to limit the wider implementation of many synthetic approaches that have been reported. Firstly, mechanistic understanding remains undeveloped. In many studies, proposed catalytic pathways are predominantly based on plausible reaction sequences, rather than rigorous kinetic, spectroscopic, or computational studies. Secondly, catalyst recyclability is often evaluated over a limited number of cycles, making it challenging to assess long-term stability and industrial feasibility. Thirdly, many reports focus on model substrates with relatively narrow substrate scopes, which may lead to an overestimation of the generality of the method. Finally, the increasing complexity of certain nanocatalysts and multifunctional catalytic materials poses questions regarding the costs, scalability, and sustainability of catalyst preparation. A critical examination of the available catalytic systems suggests that there is currently no single strategy that meets all the requirements of an optimal imidazole synthesis process. Catalyst-free processes offer operational simplicity and avoid contamination concerns, whereas transition metal catalysts often provide improved catalytic efficiency and tolerance for a range of substrates. Nanocatalysts offer high activity combined with catalyst recovery, while organocatalytic and bio-based processes offer attractive environmental benefits. As a result, the selection of an appropriate approach should be guided not only by yield but also by factors such as sustainability, catalyst availability, scalability, and downstream process requirements.

Future research should shift its focus from the development of more complex catalytic materials to establishing general design principles for sustainable imidazole synthesis. Special attention should be paid to bio-based catalysts, deep eutectic solvents, catalyst-free methods, continuous flow synthesis, photocatalytic and electrocatalytic reactions, as well as integrating computational chemistry and machine learning-assisted catalyst design. Additionally, systematic benchmark studies using standardized green chemistry metrics could facilitate more objective comparisons between different catalytic systems and help identify truly sustainable synthetic methods.

Overall, the field of imidazole synthesis is undergoing a shift from traditional, efficiency-driven approaches to more holistic and sustainable strategies. The continued integration of green chemistry principles, mechanistic understanding, and scalable process design is anticipated to play a pivotal role in the development of next-generation synthetic methodologies for imidazole derivatives. This will increase their accessibility for use in areas such as medicinal chemistry, materials science, agrochemicals, and other related fields.

## Figures and Tables

**Table 1:** Organocatalyst and bio-based catalysts for the synthesis of imidazole derivatives.

Product	Catalyst Type	Catalyst (g)	Key Advantages	Solvent, Temp. (°C)	Time	Yield Range (%)	Ref.
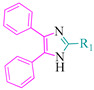	Organic acid catalysts	Citric acid (0.04 g)	Low toxicity, inexpensive, environmentally benign	Free, 130	2–8 h	80–98	[75]
		Lactic acid (1 g)	Low toxicity, inexpensive, biodegradable, environmental benign	Free, 160	3 h	62–92	[76]
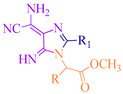	Amino acid catalysts	Glycine (1 g)	Low toxicity, high biocompatibility biodegradable, environmental benign, inexpensive,	THF, MeCN, r.t.	15–24 h	35–62	[77]
		Alanine (1 g)	Low toxicity, high biocompatibility biodegradable, environmental benign, inexpensive,	MeCN, r.t.	25	57	[77]
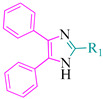	Natural product catalysts	Lemon peel powder (1 g)	Low toxicity, biodegradable, recyclable	EtOH, r.t.	45–60 min	71–91	[78]
		Lemon juice (8 mL)	Inexpensive catalysts, easy work-up, mild reaction conditions	Free, r.t.	2–9 h	80–92	[79]
		*Vitis vinifera* L. juice (8 mL)	Inexpensive catalysts, easy work-up, mild reaction conditions	Free, r.t.	1.5–8 h	81–92	[79]
		*Cocos nuci-fera* L. juice (8 mL)	Inexpensive catalysts, easy work-up, mild reaction conditions	Free, r.t.	1–7	78–89	[79]

**Table 2:** Nanostructured catalysts reported for multicomponent imidazole synthesis.

Product	Catalyst	Catalyst Composition (g)	Solvent, Temp. (°C)	Time (min)	No. of Recycle	Yield Range (%)	Ref.
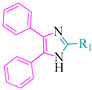	Magnetic nanoparticles	BiFeO_3_ nanoparticles (5 mg)	Free, 80	10–20	5	88–98%	[109]
		Fe_3_O_4_-based catalysts (5 mg)	MeOH, EtOH, ultrasonic irradiation, r.t.	5–20	10	82–97	[110]
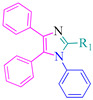	Magnetic nanoparticles	FeCeOx-based catalysts (20 mg)	MeOH, EtOH, H_2_O, 60–80	120–140	4	69–98	[111]
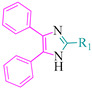	Metal oxide nanocatalysts	Cr_2_O_3_ nanoparticles (2.5 mg)	H_2_O, microwave irradiation (400 W)	6	7	92–97	[112]
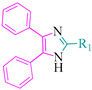	Carbon nanomaterials	Graphene oxide, (10 mg)	EtOH, ultrasonic irradiation, 110	30–55	6	74–94	[100]
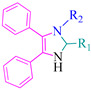	Polymer-supported catalysts	Chlorodeoxy-cellulose-based catalyst (5 mg)	EtOH, ultrasonic irradiation 80	30	5	90–97	[93]

**Table 3:** Comparison of major catalytic strategies for imidazole synthesis.

Catalytic System	Typical Catalysts/Media	Key Advantages	Limitations	Refs.
Catalyst-free systems	Thermal activation, microwave irradiation, ultrasound	Simple protocols, no catalyst contamination, environmentally benign	Sometimes requires higher temperature or longer reaction times	[50,124]
Organocatalysis	Organic acids (ascorbic acid, citric acid, glutamic acid, mandelic acid), amino acids	Low toxicity, inexpensive catalysts, compatible with green solvents	Moderate catalytic activity in some cases	[125,126]
Transition metal catalysis	Cu, Fe, Mn, Ti, lanthanide salts	High yields, broad substrate scope, efficient activation of carbonyl compounds	Possible metal contamination, catalyst recovery issues	[127,128]
Nanostructured catalysts	Magnetic Fe_3_O_4_ nanoparticles, metal oxides, graphene-based catalysts	High surface area, improved catalytic activity, recyclability	Catalyst preparation may require additional steps	[129,130,131]
Ionic liquids	Imidazolium-based ILs, specific ILs	Dual role as solvent and catalyst, recyclable, low vapor pressure	Higher cost and sometimes difficult purification	[132,133]

**Table 4:** Applications of imidazole derivatives in different research fields.

Application Area	Example Biological/Functional Activity	Compound	Refs.
Antimicrobial agents	Antibacterial and antifungal activity	Tetrasubstituted imidazole derivatives	[149,150]
Anticancer agents	Inhibition of tumor cell proliferation	Different imidazole derivatives	[151,152]
Anti-inflammatory agents	Enzyme inhibition (COX-2), protein inhibition	Tetrasubstituted imidazole derivatives	[153,154]
Corrosion inhibitors	Protection of steel and metal alloys	Trisubstituted imidazole derivatives	[155,156]
Functional materials	Ionic liquids, drug delivery, ligands, OLEDs	Different imidazole derivatives	[157,158,159]
Catalysis	Catalyst for aza-Michael addition	Imidazole	[160]
Coordination chemistry	Metal chelate affinity chromatography (IMAC), fluorescence-assisted coloration	Different imidazole derivatives	[161]

## Data Availability

The datasets used and/or analyzed during the present study are available from the corresponding author on reasonable request.
